# Nontypeable *Haemophilus influenzae* Induces Sustained Lung Oxidative Stress and Protease Expression

**DOI:** 10.1371/journal.pone.0120371

**Published:** 2015-03-20

**Authors:** Paul T. King, Roleen Sharma, Kim O’Sullivan, Stavros Selemidis, Steven Lim, Naghmeh Radhakrishna, Camden Lo, Jyotika Prasad, Judy Callaghan, Peter McLaughlin, Michael Farmer, Daniel Steinfort, Barton Jennings, James Ngui, Bradley R. S. Broughton, Belinda Thomas, Ama-Tawiah Essilfie, Michael Hickey, Peter W. Holmes, Philip Hansbro, Philip G. Bardin, Stephen R. Holdsworth

**Affiliations:** 1 Monash University Department of Medicine/Monash Medical Centre, Melbourne, Australia; 2 Monash Lung and Sleep, Monash Medical Centre, Melbourne, Australia; 3 Department of Pharmacology, Monash University, Melbourne, Australia; 4 Monash Micro Imaging, Monash University, Melbourne, Australia; 5 Clinical Immunology, Monash Medical Centre, Melbourne, Australia; 6 Monash Institute of Medical Research, Melbourne, Australia; 7 School of Biomedical Sciences and Pharmacy, University of Newcastle, Newcastle, Australia; The Hospital for Sick Children and The University of Toronto, CANADA

## Abstract

Nontypeable *Haemophilus influenzae* (NTHi) is a prevalent bacterium found in a variety of chronic respiratory diseases. The role of this bacterium in the pathogenesis of lung inflammation is not well defined. In this study we examined the effect of NTHi on two important lung inflammatory processes 1), oxidative stress and 2), protease expression. Bronchoalveolar macrophages were obtained from 121 human subjects, blood neutrophils from 15 subjects, and human-lung fibroblast and epithelial cell lines from 16 subjects. Cells were stimulated with NTHi to measure the effect on reactive oxygen species (ROS) production and extracellular trap formation. We also measured the production of the oxidant, 3-nitrotyrosine (3-NT) in the lungs of mice infected with this bacterium. NTHi induced widespread production of 3-NT in mouse lungs. This bacterium induced significantly increased ROS production in human fibroblasts, epithelial cells, macrophages and neutrophils; with the highest levels in the phagocytic cells. In human macrophages NTHi caused a sustained, extracellular production of ROS that increased over time. The production of ROS was associated with the formation of macrophage extracellular trap-like structures which co-expressed the protease metalloproteinase-12. The formation of the macrophage extracellular trap-like structures was markedly inhibited by the addition of DNase. In this study we have demonstrated that NTHi induces lung oxidative stress with macrophage extracellular trap formation and associated protease expression. DNase inhibited the formation of extracellular traps.

## Introduction

Nontypeable *Haemophilus influenzae* (NTHi) lacks a capsule (which distinguishes it from typeable forms such as HiB) and is present as a commensal in the pharynx of healthy adults. It may move down into the lower respiratory tract where it is the most common bacterium isolated in subjects with chronic bronchitis and chronic obstructive airway disease (COPD) and is an important cause of pneumonia [1]. It is also prevalent in bronchiectasis, cystic fibrosis and upper respiratory tract conditions including otitis media, conjunctivitis, tonsillitis and sinusitis. NTHi has the ability to invade extensively into the lung parenchyma in a variety of end-stage lung diseases including COPD, cystic fibrosis and interstitial lung disease [2, 3]. Despite its prevalence, the role of NTHi in lung disease has not been well defined. It has been described as “an innocent bystander” [4] but there is increasing evidence that it causes lung inflammation particularly in the context of COPD [5–7].

Chronic obstructive pulmonary disease is characterised by chronic inflammation that persists after smoking cessation. Two key processes that contribute to lung pathology in COPD are 1), oxidative stress and 2), protease imbalance [8–11]. Oxidative stress is characterised by the excessive production of reactive oxygen species (ROS) which cause local tissue damage [12, 13]. Protease imbalance leads to digestion of lung tissue by proteases such as elastase and is particularly important in the pathogenesis of emphysema. A relatively new discovery is the production of extracellular traps by phagocytic cells, principally neutrophils [14, 15]. These extracellular traps express substances such as proteases on their surface and their formation is generally dependent on ROS production.

We hypothesized that NTHi has a role in driving lung oxidative stress and in activating phagocyte extracellular trap formation with protease co-expression, and this may represent a new therapeutic target in chronic lung inflammation. We measured ROS production in in vivo mouse lung tissue and in human lung tissue in response to NTHi. We assessed the effect of this bacterium on extracellular trap formation and protease production in neutrophils and macrophages and if this process could be inhibited by the addition of DNase.

## Materials and Methods

### Study subjects

We recruited patients who were having a bronchoscopy to investigate respiratory symptoms at Monash Medical Centre/Monash Health. Bronchoalveolar lavage (BAL) of the right middle lobe was used to obtain lung cells. We also studied immune responses in human-lung cell lines and peripheral blood neutrophils. The study was approved by the Monash Health research ethics committee; and all patients provided written informed consent. For animal experiments this project was approved by the University of Newcastle animal ethics committee.

### Bacteria

Nontypeable *Haemophilus influenzae* is a bacterium that has considerable diversity with a variety of different subtypes [1]. We therefore used a variety of different strains to obtain representative results. We used a killed, pooled NTHi preparation and three distinct live strains of NTHi for the experiments [16, 17].

### Flow cytometry

We used a previously described method to measure intracellular ROS production in live cells [18]. Specimens (BAL macrophages, lung fibroblasts/epithelial cells or neutrophils) were incubated with or without NTHi for one hour. The fluorescent dye Dihydrorhodamine 123 (DHR) was also added to specimens. Specimens were run on a flow cytometer and the intensity of DHR cleavage-induced fluorescence was measured to define ROS production. To further define phagocytic cell characteristics, antibodies for CD14 (monocyte/macrophage marker) and human leukocyte antigen (HLA)-DR and CD209 (M1 and M2 macrophage markers) were added at the start of incubation as previously described [19]. Flow cytometry was also used to measure expression of matrix metalloproteinase-12 (MMP12).

### Fluorescence microscopy

To measure intracellular ROS production over time we used a fluorescence microscopy method. BAL macrophages were seeded onto 96-well plates for 1–3 days. Wells were then washed and bacteria and DHR added. Specimens were analyzed using live-cell imaging fluorescence microscopy. Hourly measures of DHR fluorescence as a measure of ROS production were done over a 17-hour time period. This method was also used to measure expression of phagocyte extracellular traps and co-expressed proteases.

### Chemiluminescence

To measure production of extracelluar ROS by live cells, chemiluminescence was used as previously described [20]. BAL macrophages were seeded onto 96-well plates for 1–3 days. Wells were then washed and bacteria added for one hour. The production of ROS was then assessed by chemiluminescence. To measure whether ROS production was extracellular, the inhibitor SOD was added to a proportion of wells.

### Mouse lung tissue

To measure lung oxidative stress to NTHi in vivo; we utilized 3-nitrotyrosine immunofluorescence (3-NT) from lung tissue sections taken from control uninfected mice and mice infected with NTHi [21]. Six eight- week old BALB/c female mice were intratracheally (IT) innoculated with 5x10^5^ colony-forming units (CFU) of non-typeable *Hi* (NTHi-289 in 30ul sterile PBS) under Alfaxan anaesthesia (12.5mg/kg, IV). Sham groups received PBS. Five days later mice were euthanised with 325mg/kg sodium pentobarbitone (IP). The left lung was removed, fixed in formalin, then imbedded in paraffin, sectioned (4–6μm), and stained with relevant stains. Image J software was used to analyze differences in fluorescence.

### Statistical analyses

Characteristics of the subjects were summarized using descriptive statistics. Comparison between control and stimulated groups was done using paired or unpaired-testing with parametric or non-parametric methods as appropriate. Between-group differences were analyzed by one-way analysis of the variance (ANOVA). A p value of less than 0·05 was considered to indicate statistical significance.

(Additional methodological details are provided in S1 Text).

## Results

### Characteristics of subjects

To study the BAL macrophage response to NTHi we recruited 121 subjects. Characteristics of the subjects are summarized in Table 1. We also recruited 15 healthy controls to study neutrophil responses to NTHi. Human cell tissue lines for fibroblasts (*n* = 8 subjects) and epithelial cells (*n* = 8 subjects) were obtained from bronchial biopsies. Further details about the subjects are listed in the Supporting Information.

**Table 1:** Baseline characteristics of the BAL patients.

Characteristic	Study group			
	Entire group	No definable lung disease	COPD	Other inflammatory lung disease
**Number of subjects**	121	34	40	47
**Age (mean & SD)**	59±16	59±12	64±16	54±17
**Sex**				
Male	60	15	25	20
Female	61	19	15	27
**Significant smoking history**				
Numbers of patients	42	3	31	8
Pack years (in smokers)				
Median	30	20	38	23
IQR	20–45	15–30	25–67	16–45
**Differential cell count (% of cells)**				
Macrophages				
Median	89	91	84	89
IQR	74–93	79–94	54–94	74–93
Lymphocytes				
Median	9	9	8	9
IQR	6–17	5–16	5–15	6–20
Neutrophils				
Median	1	1	3	1
IQR	1–6	1–5	1–13	1–4
Eosinophils				
Median	0	0	0	0
IQR	0–1	0–1	0–1	0–1
**Spirometry**				
FEV_1_ (% of predicted value)				
Mean & SD	91±23	105±18	71±18	101±17
FVC (% of predicted value)				
Mean & SD	97±20	104±17	92±15	97±24
FEV_1_/FVC				
Mean & SD	72±11	81±3	61±8	80±5

### NTHi induces lung oxidative stress mediator production

**Fig 1:**
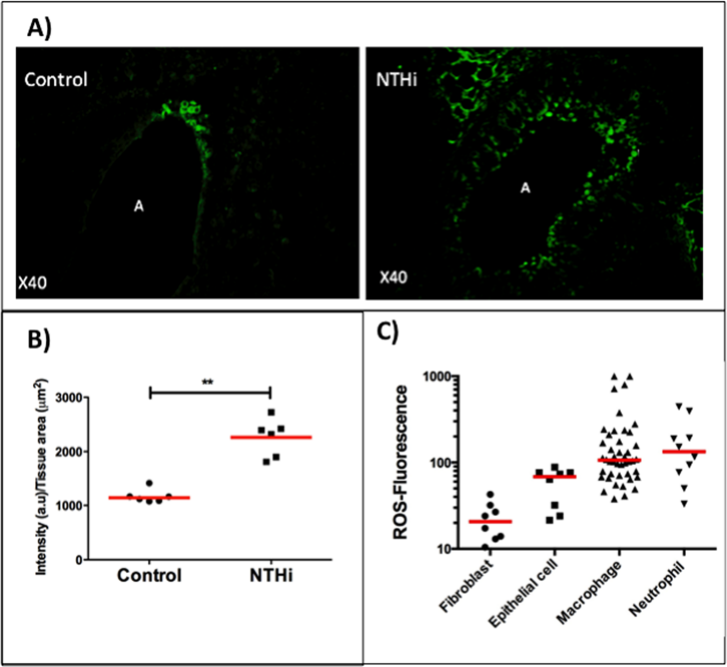
Nontypeable *Haemophilus influenzae* generates the production of oxidative stress mediators in lung tissue. Panel A shows immunoreactivity for 3-nitrotyrosine (a toxic oxidative stress product) in lung tissue from uninfected (control) and NTHi-infected mice and Panel B shows intensity of 3-nitrotyrosine immunoreactivity which is significantly higher in NTHi group (control 1141 (1084–1227) NTHi 2538 (1877–2497)), (n = 6, p = 0·002) (A = airway). Panel C shows the ROS production in human cells by; lung fibroblasts (n = 8), lung epithelial cells (n = 8), lung macrophages (n = 44) and blood neutrophils (n = 10) as measured by flow cytometry of DHR cleavage-induced fluorescence. There is a progressive increase in ROS fluorescence between the cell types (fibroblasts 21 (13–31), epithelial cells 69 (26–76), macrophages 107 (71–201) and neutrophils 134 (70–242)) with the highest levels in the phagocytes (*P* < 0·001, ANOVA); with significant between-group differences between 1), the fibroblasts and i) epithelial cells ii), macrophages and iii), neutrophils (all p<0·05) and 2), the epithelial cells and i), macrophages and ii), neutrophils (all p<0·05).

We studied samples of mouse lung that had been infected in vivo with NTHi five days previously and found prominent tissue production of 3-nitrotyrosine (a potent oxidant), with significantly higher levels of fluorescence in infected mice when compared to uninfected mice (n = 6, p = 0.002) (Fig. 1A and B). We found widespread staining throughout the lung for 3-NT suggesting the involvement of multiple cell types.

We then studied the in vitro expression of intracellular ROS from human cells in samples obtained from BAL, peripheral blood, and lung-fibroblast and lung-epithelial cell lines. Cells were stimulated with NTHi and ROS production assessed by DHR fluorescence, was measured by flow cytometry. Initial experiments with BAL phagocytes demonstrated that both killed and live NTHi enhanced ROS production (S1 Fig in the supplement). The surface marker CD14 was used to define the macrophages in the BAL phagocyte population. Live NTHi stimulation induced significant increases in ROS production in all four cell types (lung fibroblasts n = 8 p = 0.008, lung epithelial cells n = 8 p = 0.008, lung macrophages n = 44 p<0.001 and neutrophils n = 10 p = 0.024) when compared to control (non-stimulated) samples (S2 Fig).

A large variety of different cell types contain NOX NADPH oxidases which are responsible for the production of ROS [22]. The most active form is the NOX2 oxidase which is present at highest levels in the phagocytic cells and is used as a primary mechanism to kill phagocytosed pathogens. There was a progressive increase in ROS production between the different cell types (p<0.001 ANOVA) and levels of ROS production to live NTHi were significantly higher in the phagocytes when compared to the fibroblast and epithelial cell lines (Fig. 1C).

### NTHi induces sustained and extracellular production of ROS by lung macrophages

**Fig 2:**
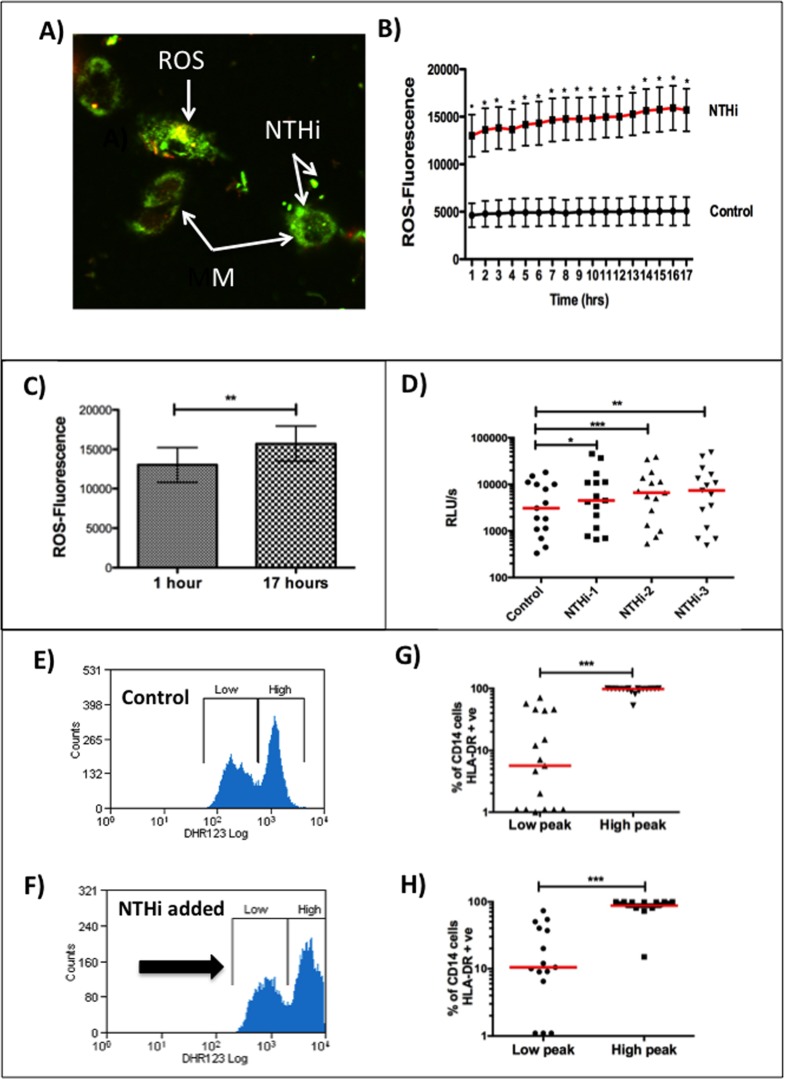
Response by human bronchoalveolar macrophages to nontypeable *Haemophilus influenzae*. Panel A shows adherent BAL macrophages with staining for NTHi and production of ROS fluorescence in macrophages infected with NTHi by confocal microscopy. Panel B shows the ROS production in control (uninfected) macrophages and NTHi-infected macrophages over a 17 hour time-period (n = 15 subjects for each time-point) measured by confocal microscopy. At each one-hour time-point ROS production is significantly higher in the NTHi group (p<0·01). Panel C shows the increase over 17 hours in ROS production (1 hour 13012±2214, 17 hours 15175±2247)), (n = 15, p = 0·009). Panel D shows upregulation of ROS as measured by the effect of three different strains of NTHi using chemiluminescence (control 5937 (1839–10855), NTHi-1 8193 (3569–15538), NTHi-2 8953 (5105–17111), NTHi-3 10093 (4136–20848)), (RLUs = reactive light units/second). The chemiluminescence predominantly measures extracellular ROS production. Panels E and F demonstrates an examples of the presence of high and low-producing ROS fluorescence populations in control (unstimulated) and NTHi-stimulated macrophages from a patient (using flow cytometry). The high-producing ROS populations have significantly increased surface expression of the M1 marker HLA-DR (p<0·001): Panel G shows the unstimulated (baseline) macrophage population (low peak 6 (1–45), high peak 98 (94–99)), (n = 17), whilst Panel H shows the NTHi-stimulated macrophage population (low peak 11 (7–40), high peak 95 (80–100)), (n = 15).

As macrophages are the first cells involved in host defence against airway bacterial infection and express high levels of ROS to NTHi, we investigated their function in more detail. To assess the change in intracellular ROS over time we studied adherent BAL macrophages using fluorescence microscopy in an ex vivo assay. Cells were incubated with multiple strains of live NTHi and ROS measured by intensity of DHR fluorescence (Fig. 2A). Over the 17-hour period, the level of ROS was significantly increased compared to control at each hourly time-point (n = 15 subjects, p<0.01) (Fig. 2B). Over the 17-hour time-period there was a significant increase in ROS production in macrophages stimulated with NTHi (n = 15 subjects, p = 0.009) (Fig. 2C). Levels of ROS prouduction increased more with the confocal technique (three-fold with NTHi) than with the flow cytometry method (30–40% with NTHi). This is likely to be to the flow cytometry method in which cells were directly obtained from BAL and then put on rotation thus increasing their baseline activity state (in contrast to the confocal method in which cells were rested overnight before assays were performed).

The flow cytometry and confocal microscopy methods measure intracellular ROS production. Chemiluminescence was used to measure extracellular ROS production by alveolar macrophages stimulated with NTHi and demonstrated a significant increase (n = 12 subjects, p<0.001 ANOVA) with significant between-group differences (p<0.05) (Fig. 2D). Extracellular ROS production was significantly inhibited by the addition of SOD, confirming predominant extracellular origin (S4 Fig). Therefore NTHi produces a sustained extracellular production of ROS that increases over time and could potentially cause local tissue damage.

As NTHi is a bacterium with considerable diversity we compared results of strain 1 of NTHi which was used for the majority of the experiments, with two other well-defined strains of NTHi. We found similar changes in ROS production with all three live strains of NTHi (S1–S3 Tables and S1, S3, and S4 Figs).

### Polarized macrophage responses

Using the flow cytometry method we noted a bimodal distribution of ROS production in alveolar macrophages in most subjects (29/44 subjects or 66%) at rest. With stimulation with NTHi this bimodal distribution persisted in most patients (26/29 or 90%) and both populations had significantly increased ROS production after NTHi stimulation (Figs. 2E, 2F, and S5). This result suggests that in many subjects there are two populations of ROS producing macrophages; a low and a high population. To our knowledge this is a novel finding. Previous literature has established that there are two functional forms of macrophages; designated as M1 and M2 subtypes [23]. The M1 subtype is involved in active inflammation whilst the M2 subtype is involved in the healing immune response. To assess whether the high-producing ROS cells expressed HLA-DR an established marker of M1 phenotype, a specific antibody for HLA-DR and CD209 a marker of M2 phenotype, were added and their expression measured by flow cytometry. The high-ROS group had significantly increased expression of HLA-DR compared to the low-ROS group (Figs. 2G and H, and S5). In contrast there were no differences in CD209 expression (with low expression of <1%) between groups.

### NTHi stimulates the production of phagocyte extracellular traps and protease expression

The production of ROS has a number of pro-inflammatory effects, one of which is the formation of extracellular traps. Extracellular traps are extensions of DNA (chromatin) from the cell with the co-expression of granule proteases and have been best described in the context of neutrophils (neutrophil extracellular traps (NETs)) [15].

**Fig 3:**
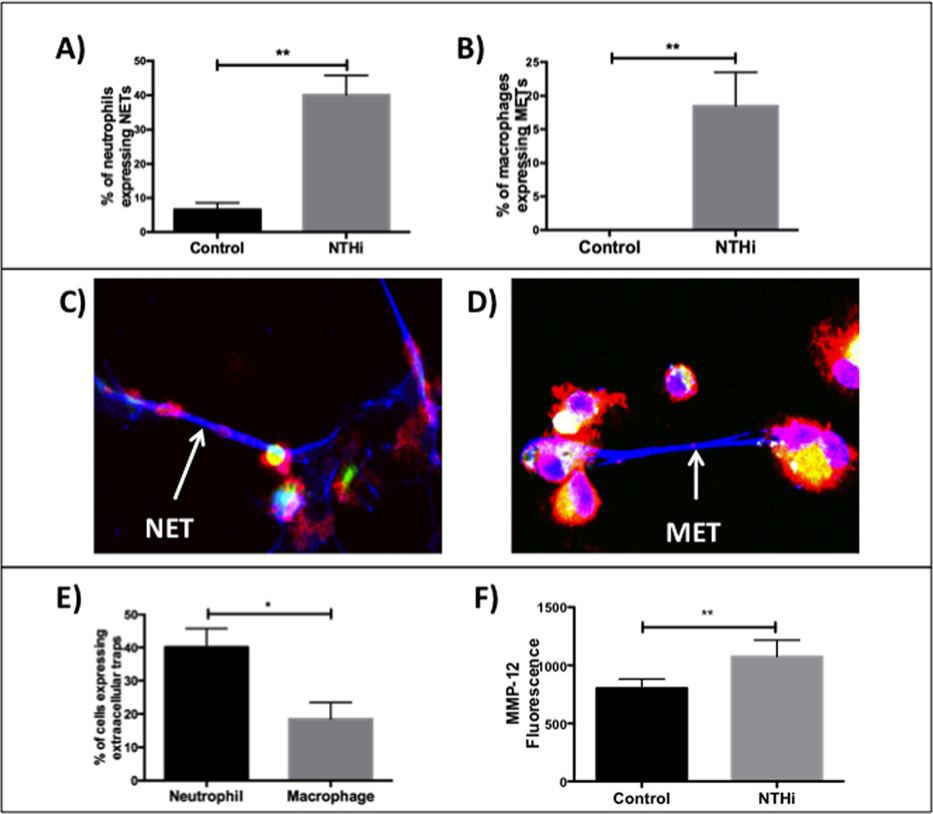
Production of phagocyte extracellular traps and proteases in response to nontypeable *Haemophilus influenzae*. Panel A shows the % of neutrophils expressing neutrophil extracellular traps (NETs), (control 7 ±2, NTHi 40±6)), (n = 7, p = 0·002)) and Panel B shows the % of BAL macrophages expressing macrophage extracellular traps-like structures (METs), (control 0, NTHi 19±5), (n = 8, p = 0·008); after stimulation with NTHi compared to control. Panel C shows neutrophils producing NETs with co-expression of neutrophil elastase (arrow). Panel D shows macrophages producing a MET with co-expression of macrophage metalloproteinase-12 (arrow). Panel E shows that neutrophils (40±6, n = 7) have a higher expression of extracellular traps compared to macrophages (19±5, n = 8) (p = 0·013). Panel F; NTHi increases surface expression of MMP12 by BAL macrophages as measured by flow cytometry (control 804±78, NTHi 1075±142)), (n = 8, p = 0·005).

We stimulated peripheral blood neutrophils with NTHi and measured their production of NETs. In healthy controls we found that NTHi induced NET formation with surface expression of neutrophil elastase (n = 7 subjects, p = 0.002) (Fig. 3A and C).

One recent study has described that macrophages may also express extracellular traps (designated as MET-like structures) [24]. We stimulated BAL macrophages from eight subjects and found that all subjects produced MET-like structures with the expression of MMP12 (n = 8 subjects, p = 0.008) (Fig. 3B and D). MMP12 is known to be a key mediator involved in the development of emphysema [25, 26]. The proportion of cells that produced extracellular traps was higher in the neutrophils when compared to macrophages (p = 0.013) (Fig. 3E). We also used flow cytometry to measure MMP12 surface expression in eight subjects’ BAL macrophages and found that NTHi induced a significant increase in MMP12 expression (p = 0.005) (Fig. 3F). Staining for NETs/METs is shown in more detail in S6 Fig.

**Fig 4:**
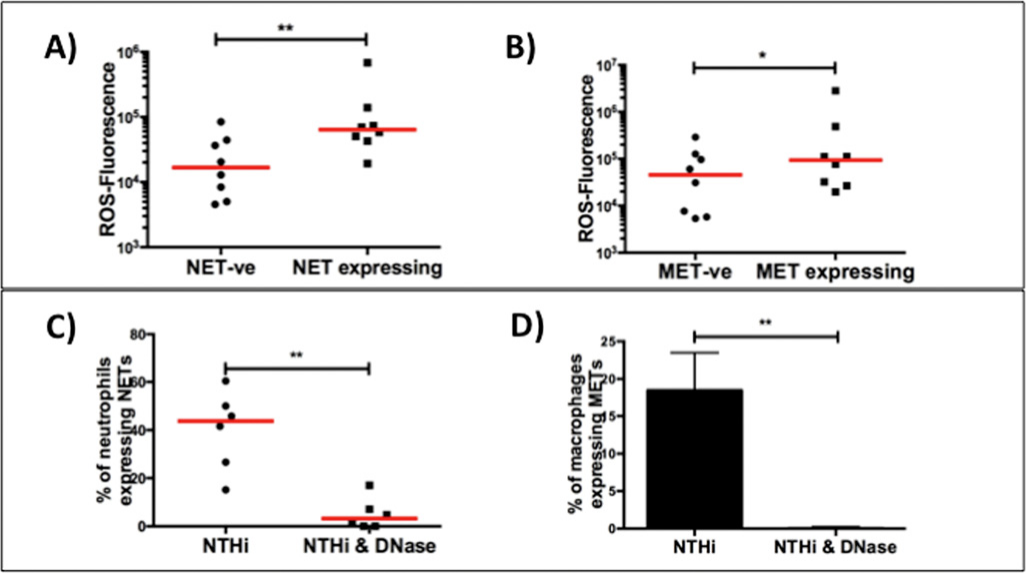
Production of extracellular traps is associated with ROS production and is inhibited by DNase. Panels A shows that neutrophils expressing NETs have a four-fold increase in ROS fluorescence compared to NET negative (-ve) cells (NET-ve 16 706 (5879–42 332), NET+ve 64 146 (44 738–122921)), (n = 7, p = 0·008). Panel B shows that macrophages expressing MET-like structures have a two-fold increase in ROS fluorescence compared to MET negative (-ve) cells (MET-ve 45 644 (6257–118907), MET+ve 93 636 (28 113–392 973)), (n = 8, p = 0·023). Panels C and D demonstrate inhibition of NET (NTHi 44 (24–53), NTHi & DNase 3 (0–10)), (n = 6, p = 0·004) and MET-like structure (NTHi 19±5, NTHi & DNase 0.1±0.1)), (n = 8, p = 0·008) formation by the addition of DNase.

ROS production is known to be a key factor in the production of extracellular traps [15] and we found that the levels of ROS were significantly higher in neutrophils expressing NETs (p = 0.008) and macrophages expressing MET-like structures (p = 0.023) when compared to cells that did not express extracellular traps (Fig. 4A and B). To further define the association between ROS and MET-like structures formation we added the ROS inhibitor apocynin to macrophages incubated with NTHi. This resulted in downregulation of MET-like structures expression (n = 7 subjects, p = 0.03) (S7 Fig).

### DNase inhibits phagocyte extracellular trap formation

DNase cleaves extracellular DNA which improves mucociliary function and it is used as a standard maintenance therapy in patients with cystic fibrosis (CF) to improve lung function and symptoms [27, 28]. It has also been recognised to reduce markers of inflammation including neutrophil proteases and NET formation. We found that the addition of DNase caused marked inhibition (> than 90%) of both NET (n = 6 subjects, p = 0.004) and MET-like structures formation (n = 8 subjects, p = 0.008) after NTHi stimulation (Fig. 4C and D, S4 Table and S8 Fig).

### Patient subgroups

The 121 patients who had had BALs were divided into three separate subgroups. Thirty-four subjects were classified as having no definable lung disease, 40 subjects had COPD and 47 subjects as having other inflammatory lung disease (Table 1). When the subgroups were analyzed for ROS production and macrophage killing function; there were no significant differences between the three groups (S9 Fig).

## Discussion

In this study we examined the effect of the prevalent bacterium *nontypeable Haemophilus influenzae* on lung inflammation. Our investigations demonstrated that NTHi infection induces the sustained and extracellular production of reactive oxygen species. Levels of ROS production were highest in neutrophils and alveolar macrophages. The alveolar macrophages had a bimodal prodution of ROS. We found that the generation of ROS was associated with the production of neutrophil and macrophage extracellar traps. These NETs and METs expressed the proteases; neutrophil elastase and macrophage metalloproteinase-12 respectively, on their surface. The addition of DNase markedly inhibited the production of the extracellular traps.

NTHi is an opportunistic mucosal pathogen that can move down into the lung in a variety of chronic lung diseases. It is the most prevalent bacterium isolated from lower respiratory tract in patients with COPD; both in terms of acute exacerbations and chronic colonization. NTHi is also prominent in a variety of other respiratory conditions. Unusally for a common respiratory bacterium it has the ability to be able to invade extensively into the lung parenchyma [2, 3]. The role of NTHi in inducing inflammation in the lower respiratory tract has been controversial [4] but recent studies highlight its important role in this regard.

The production of ROS by phagocytes is a primary mechanism used to kill phagocytosed pathogens and deficiency of this mechanism results in life-threatening infections as occurs in chronic granulomatous disease (CGD). As NTHi is a common bacterium found in lung disease it would be expected to have an effect on ROS production. Surprisingly there appears to be negligible published literature on this topic. Naylor et al described that NTHi induces an increase in ROS production in peripheral blood neutrophils [29]. However NTHi does have mechanisms including the production of catalase and SOD, to help it combat the effects of oxidative stress [30]. As NTHi is almost exclusively a respiratory pathogen it suggests that it has developed these protective mechanisms as it is exposed to oxidative stress in the respiratory tract.

Oxidative stress has been clearly established to be one of the key pathogenic processes in COPD [8]. It has effects both in terms of local tissue damage and the systemic inflammatory state present in COPD. Smoking is the primary factor associated with oxidative stress [9]. However despite smoking cessation there is persistent inflammation and lung damage [31, 32]. The factors that drive this inflammation have not been well defined although bacterial infection has been proposed to be a contributing factor [8]. We found that NTHi-induced oxidative stress across the different subgroups (i.e. those with no definable lung disease, COPD patients and other inflammatory lung disease) suggesting that this mechanism may have implications for lung conditions in addition to COPD such as bronchiectasis and cystic fibrosis [33, 34]. Whilst antioxidant therapy has not been generally been successful, the specific targeting of NTHi may have potential to inhibit lung ROS production.

One of the effects of ROS production is to induce the formation of neutrophil extracellular traps [15]. We found that NTHi induced the formation of NETs with expression of neutrophil elastase which has been described as an effect in bacterial infections such as with *Staphylococcus aureus* [14]. Juneau at al have described that NTHi induces NETs in human blood neutrophils [35]. These neutrophil extracellular traps have an important role in defence against infection but they also express mediators which are potentially damaging to local tissue (such as elastase). It has been recently reoognised that other cells may express these extracellular traps, including a report of this occuring in human macrophages [24]. The formation of extracellular traps in macrophages is not yet well established in the literature. We used criteria that have previously been used to define NETs (extracellular expression of chromatin and proteases, driven by ROS production and inhibited by DNase) to define the effect on what we have designated as macrophage extracellular trap-like structures. We demonstrated that NTHi induced the formation of MET-like structures and this was associated with expression of MMP12. The proteases NE and MMP12 have been clearly established to be key factors in the development of protease imbalance and emphysema. Therefore the production of NETs and METs by phagocytes in response to NTHi infection has potential implications for the development of emphysema. This is most likely to be relevant in patients who have chronic infection with this bacterium, which could potentially result in sustained production of extracellular traps.

We found that the production of NETs and MET-like structures was markedly inhibited by the addition of DNase. DNase is standard maintenace therapy to improve sputum clearance and reduce bacteria-induced inflammation in cystic fibrosis [27]. There are potential issues with its use though, in that it can be damaging to the lung when used on a long-term basis and it has been associated with adverse outcomes in non-CF bronchiectasis when used as a long-term maintenance therapy [36]. Our work suggests that there may be a role for DNase in the prevention of NTHi-induced NET/MET-like structures formation with its associated protease expression. This may be most likely to be effective as a short-term agent in the context of NTHi-induced exacerbations of lung disease.

Nontypeable *Haemophilus influenzae* is the most common bacterium isolated in subjects with COPD. However it is also a major bacterium associated with a number of other inflammatory respiratory conditions including pneumonia, bronchitis, bronchiectasis/cystic fibrosis, otitis media and sinusitis. In addition it has also been shown to extensively invade the lung in a variety of end-stage pulmonary conditions including COPD, cystic fibrosis and interstitial lung disease. Therefore this bacterium may have a potentially important role in a wide variety of lung conditions in addition to COPD. The primary aim of this study was to demonstrate that NTHi induces lung oxidative stress and this is associated with MET-like structure formation and protease expression. As a secondary end-point we were interested in whether these findings could be demonstrated in different subgroups. We found that in the three subgroups of 1), no definable lung disease 2), COPD and 3), other inflammatory lung disease, that NTHi induced ROS and was associated with the formation of MET-like structures. These three subgroups were quite heterogeneous with a variety of different lung conditions, smoking history, medication use and respiratory infection. There may well be between-group differences in oxidative stress and MET-like structure formation to NTHi but the current study did not aim to detect whether such differences may be present. Further studies to investigate potential between-group differences would be of interest.

In this study we have demonstrated that NTHi induces lung oxidative stress with phagocyte extracellular trap formation and associated protease expression. Specific targetting of this bacterium is likely to reduce these pathogenic lung processes, The results also emphasize the potential role of DNase as a therapeutic option in this context.

## Supporting Information

S1 Dataset ZIP file showing images of MET formation (control, NTHi and NTHi/Dnase) of the 8 subjects (S1–8 Dataset). The images are the raw picutres taken from the confocal microscope. They can be opened with NIKON software using the attached link: http://www.nikoninstruments.com/Products/Software/NIS-Elements-Advanced-Research/NIS-Elements-Viewer.
(ZIP)

S2 Dataset ZIP file showing images of MET formation (control, NTHi and NTHi/Dnase) of the 8 subjects (S1–8 Dataset). The images are the raw picutres taken from the confocal microscope. They can be opened with NIKON software using the attached link: http://www.nikoninstruments.com/Products/Software/NIS-Elements-Advanced-Research/NIS-Elements-Viewer.
(ZIP)

S3 Dataset ZIP file showing images of MET formation (control, NTHi and NTHi/Dnase) of the 8 subjects (S1–8 Dataset). The images are the raw picutres taken from the confocal microscope. They can be opened with NIKON software using the attached link: http://www.nikoninstruments.com/Products/Software/NIS-Elements-Advanced-Research/NIS-Elements-Viewer.
(ZIP)

S4 Dataset ZIP file showing images of MET formation (control, NTHi and NTHi/Dnase) of the 8 subjects (S1–8 Dataset). The images are the raw picutres taken from the confocal microscope. They can be opened with NIKON software using the attached link: http://www.nikoninstruments.com/Products/Software/NIS-Elements-Advanced-Research/NIS-Elements-Viewer.
(ZIP)

S5 Dataset ZIP file showing images of MET formation (control, NTHi and NTHi/Dnase) of the 8 subjects (S1–8 Dataset). The images are the raw picutres taken from the confocal microscope. They can be opened with NIKON software using the attached link: http://www.nikoninstruments.com/Products/Software/NIS-Elements-Advanced-Research/NIS-Elements-Viewer.
(ZIP)

S6 Dataset ZIP file showing images of MET formation (control, NTHi and NTHi/Dnase) of the 8 subjects (S1–8 Dataset). The images are the raw picutres taken from the confocal microscope. They can be opened with NIKON software using the attached link: http://www.nikoninstruments.com/Products/Software/NIS-Elements-Advanced-Research/NIS-Elements-Viewer.
(ZIP)

S7 Dataset ZIP file showing images of MET formation (control, NTHi and NTHi/Dnase) of the 8 subjects (S1–8 Dataset). The images are the raw picutres taken from the confocal microscope. They can be opened with NIKON software using the attached link: http://www.nikoninstruments.com/Products/Software/NIS-Elements-Advanced-Research/NIS-Elements-Viewer.
(ZIP)

S8 Dataset ZIP file showing images of MET formation (control, NTHi and NTHi/Dnase) of the 8 subjects (S1–8 Dataset). The images are the raw picutres taken from the confocal microscope. They can be opened with NIKON software using the attached link: http://www.nikoninstruments.com/Products/Software/NIS-Elements-Advanced-Research/NIS-Elements-Viewer.
(ZIP)

S9 Dataset A ZIP file showing effect of apocynin on extracellular chromatin expression. (ZIP)

S1 Fig ROS production by lung phagocytes. Response to killed bacteria: A) ROS production by lung phagocytes (n = 18 subjects) was significantly increased with killed NTHi antigen (control 20 (14–66), NTHi 37 (20–85)), (p = 0·002) (Wilcoxon matched-pairs rank test). B) ROS production by lung phagocytes (n = 18 subjects) was significantly increased by killed *S*. *aureus* antigen (control 26 (14–65), S. aureus 50 (29–122)), (p = 0·014) (Wilcoxon matched-pairs rank test). ROS response was similar with both bacteria. Lung phagocytes obtained from BAL were incubated with three different strains of NTHi (NTHi 1–3): C), NTHi-1 (control 50 (28–64), NTHi 68 (36–151)) (76 subjects), D), NTH-2 (control 68 (45–102), NTHi 91 (49–170)) (48 subjects) and E), NTH-3 (control 72 (57–98), NTHi 94 (71–140)) (22 subjects) all induced a significant increase in ROS (p<0·001) (Wilcoxon matched-pairs rank test). ROS production to killed NTHi (37 (20–85)), 18 subjects) was compared to live strains of NTHi-1 (68 (36–151)), 76 subjects), NTHi-2 (91 (49–170)), 48 subjects) and NTHi-3 (94 (71–140)), 22 subjects). Kruskall Wallis testing demonstrated overall significant difference (p = 0·002) and also significant increases when each live strain was compared to killed NTHi (For the phagocyte/macrophage assays, cells were obtained freshly from BAL and then put on rotation which would enhance baseline activity state).
(TIF)

S2 Fig ROS production by different cell types to live NTHi. Cells types were stimulated with NTHi strain 1 and ROS measured using flow cytometry. A) Human fibroblast cell-lines (n = 8) had increased ROS (p = 0·008) following NTHi infection (control 13 (10–18), NTHi 21 (13–31)). B) Human epithelial cell-lines (n = 8) had increased ROS (p = 0·008) following NTHi infection (control 57 (23–68), NTHi 21 (13–31)). C) Human BAL macrophages (n = 44) had increased ROS (P< 0·001) following NTHi infection (control 80 (61–142), NTHi 107 (74–200)). D) Human blood neutrophils (n = 10) had increased ROS (p = 0·024) following NTHi infection (control 69 (41–99), NTHi 133 (70–242)). Analysis done by Wilcoxon matched-pairs rank test.
(TIF)

S3 Fig ROS response to different strains of NTHi. Neutrophils and macrophages were stimulated with multiple strains of NTHi to measure response. A) Peripheral blood from ten healthy controls was stimulated with three strains of live NTHi. Neutrophils were gated on, based on forward and side scatter and low levels of CD14 staining. There was a significant change in ROS production as measured by ANOVA, (p = 0·0008) with significant increases in each of the three strains compared to control (paired t-test) (control 68±11, NTHi-1 174±44, NTHi-2 197±37. NTHi-3 176±40). Lung macrophages obtained from BAL were incubated with two other strains of NTHI (NTH1–2 and NTHi-3): B), NTHi-2 (control 94 (63–157), NTHi-2 121 (74–217)) (31 subjects) and C), NTHi-3 (control 81 (63–121), NTHi-3 117 (91–147)) (15 subjects). All induced a significant increase in ROS production (p<0·001) (Wilcoxon matched-pairs rank test). Fluorescent microscopy was used to measure production of ROS over a 17-hour period in adherent macrophages, with readings of ROS-fluorescence taken every hour. B) Macrophages were stimulated with NTHi-2 and at all times there was a significant increase in ROS compared to control (p<0·001) (n = 15). C) Macrophages were stimulated with NTHi-3 and at all times there was a significant increase in ROS compared to control (p<0·01) (n = 11). All analyses done by paired t-testing. Levels of ROS were three-fold higher with NTHi stimulation (above control) with this method; compared to the approximately 40% increase when using the flow cytometry method (this difference is likely to be due to the cells for flow cytometry method being recently obtained from BAL (i.e. same day) and then being put on rotation and thus increasing the baseline activity state; when compared with the confocal assay where cells were seeded onto plates and allowed to rest overnight before being stimulated).
(TIF)

S4 Fig Extracellular macrophage ROS production is inhibited by the addition of superoxide dismutase (SOD). Adherent macrophages from ten subjects were stimulated with NTHi or NTHi with SOD. With all three strains there was significantly reduced ROS (expressed as RLU/s) A), NTHi-1 (NTHi 6037 (3990–12 461), NTHi & SOD 1291 (869–4086)), p = 0·004 B), NTHi-2 (NTHi 7101 (5364–14 824), NTHi & SOD 891 (574–3357)), p = 0·002 and C) NTHI-3, (NTHi 9446 (6750–19 323), NTHi & SOD 616 (491–2484)), p = 0·004 (Wilcoxon matched-pairs rank test).
(TIF)

S5 Fig Both low and high-peak (ROS) populations of macrophages increase ROS production after NTHi stimulation. At baseline there were low and high-ROS producing populations of macophages (n = 26 subjects). Panel A demonstrates a significant increase in ROS production after NTHi stimulation in comparison to control (non-infected population) in the low-peak group (control 36 (21–55), NTHi 44 (27–92)), (p < 0·001). Panel B demonstrates a significant increase in ROS production after NTHi stimulation in comparison to control (non-infected population) in the high-peak group (control 143 (121–401), NTHi 163 (115–678)), (p = 0·004). Panel C shows the high-peak had a higher percentage of macrophages that express HLA-DR.
(TIF)

S6 Fig Expression of neutrophil extracellular traps (NETs) and macrophage extracellular traps (METs). The left-hand column shows NET expression. Panel A) shows merged picture with Panel B) showing staining for chromatin, Panel C) shows staining for neutrophil elastase, Panel D) shows staining for ROS, and Panel E) shows staining for histone. The right-hand column shows MET expression. Panel A) shows merged picture with Panel B) showing staining for chromatin, Panel C) shows staining for macrophage metalloproteinase-12 and Panel D) shows staining for ROS.
(TIF)

S7 Fig Effect of ROS inhibitor apocynin on MET formation. Macrophages were incubated with NTHi and apocynin was added to inhibit ROS production and the expression of METs as measured by the proportion of extracellular DNA of macrophages. Apocynin significantly decreased MET expression (NTHi 49 (34–56), NTHi & apocynin 27 (21–28)) (n = 7, p = 0·03) (Wilcoxon matched-pairs rank test).
(TIF)

S8 Fig Expression of METs after NTHi stimulation and the effect of DNase at different time-points. BAL macrophages (n = 8 subjects) were stimulated with NTHi and with/without DNase and the expression of METs measured by confocal microscopy at A), 20 minutes (control 0, NTHi 19± 5, NTHi & DNase 0.1±0.1) B), one hour (control 0, NTHi 19±5, NTHi & DNase 0.1±0.1) and C), three hours (control 0, NTHi 19±5, NTHi & DNase 0.1±0.1). At all 3 time-points there were significant differences (p<0·01) with both NTHi and DNase (paired t-testing).
(TIF)

S9 Fig Functional properties of cells in the three patient subgroups. NTHi stimulation significantly increased ROS production to NTHi in lung phagocytes (A-C) and lung macrophages (D-F) in the three subgroups: A) No definable lung disease group (control 52 (28–99), NTHi 68 (38–165)), (n = 24 subjects), p<0·001, B), other lung disease group (control 46 (27–95), NTHi 79 (33–154)), (n = 29 subjects) p<0·001 and C), COPD group (control 48 (26–77), NTHi 69 (40–119)), (n = 22 subjects) p<0·001. ROS production to NTHi in lung macrophages: D) No definable lung disease group (control 93 (62–137), NTHi 103 (76–227)), (n = 14 subjects), p<0·001, E), other lung disease group (control 68 (38–124), NTHi 103 (74–229)), (n = 17 subjects) p<0·001 and F), COPD group (control 73 (61–129), NTHi 109 (82–166)), (n = 13 subjects) p = 0·008. All analyses by Wilcoxon matched-pairs rank test. G) There were no significant differences in the levels of ROS production by lung phagocytes stimulated with NTHi (p = 0·95). H) There were no significant differences in the levels of ROS production by lung macrophages stimulated with NTHi (p = 0·91). I) There were no significant differences in the bacterial killing of NTHi-1 in the patient groups (no definable lung disease group 410 (60–610)), other lung disease group 20 (0–2050), COPD group 1020 (450–2015)) (p = 0·18). J) There were no significant differences in the bacterial killing of NTHi-2 in the patient groups (no definable lung disease group 360 (30–700)), other lung disease group 15 (0–810), COPD group 620 (350–1710)) (p = 0·14). Statistical analysis performed with one-way ANOVA and Kruskal-Wallis test.
(TIF)

S1 Table ROS production by lung phagocytes to different strains of NTHi. ROS is the measure of fluorescence induced by DHR-cleavage with results expressed as median and interquartile ranges. Statistical analysis performed using Wilcoxon matched-pairs rank test.
(PDF)

S2 Table ROS production by lung macrophages. ROS is the measure of fluorescence induced by DHR-cleavage with results expressed as median and interquartile ranges. Statistical analysis performed using Wilcoxon matched-pairs rank test.
(PDF)

S3 Table ROS production of lung macrophages measured by chemiluminescence. Chemiluminescence is expressed as reactive light units per second (RLU/s). Statistical analysis performed by one-way analysis of the variance using Friedman test.
(PDF)

S4 Table Percentage of neutrophils and macrophages expressing extracellular traps (NET or MET-like structures). The % of neutrophils or macrophages expressing extracellular traps (either as NETs or MET-like structures) as control, after NTHi stimulation and with the combined effect of NTHi and DNase (different timepoints of 20 minutes, 1 hour and 3 hours)
(PDF)

S1 Text Additional methodological information and references. (DOCX)
