# Supplementary material for: Nontypeable Haemophilus influenzae Induces Sustained Lung Oxidative Stress and Protease Expression
Source: PLoS One. 2015 Mar 20;10(3):e0120371. doi: 10.1371/journal.pone.0120371 (PMC4368769; doi:10.1371/journal.pone.0120371)
Supplement: S9 Dataset — (ZIP) [file pone.0120371.s009.zip › S9_Dataset.pptx]

## Slide 1
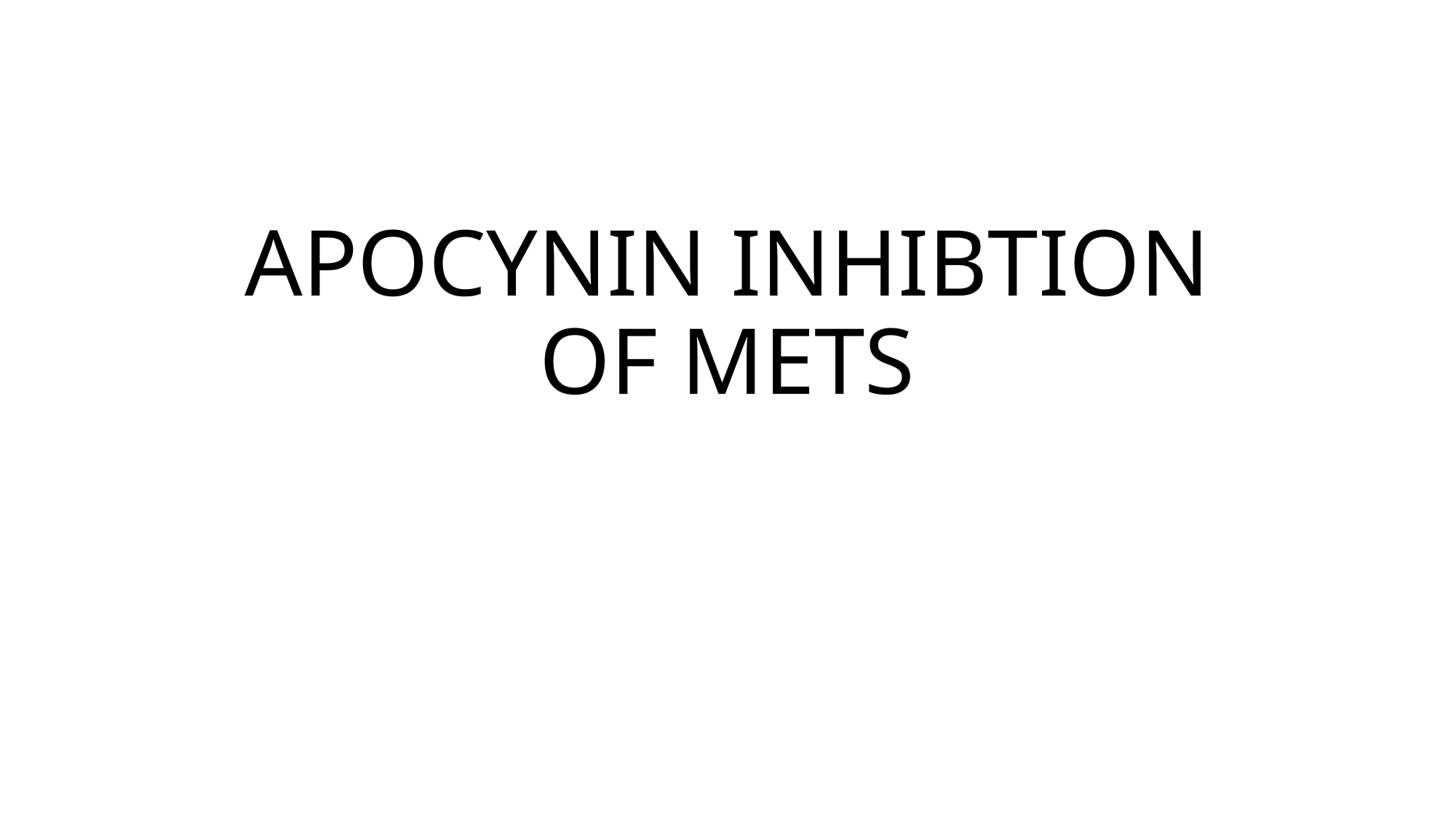

# APOCYNIN INHIBTION OF METS

## Slide 2
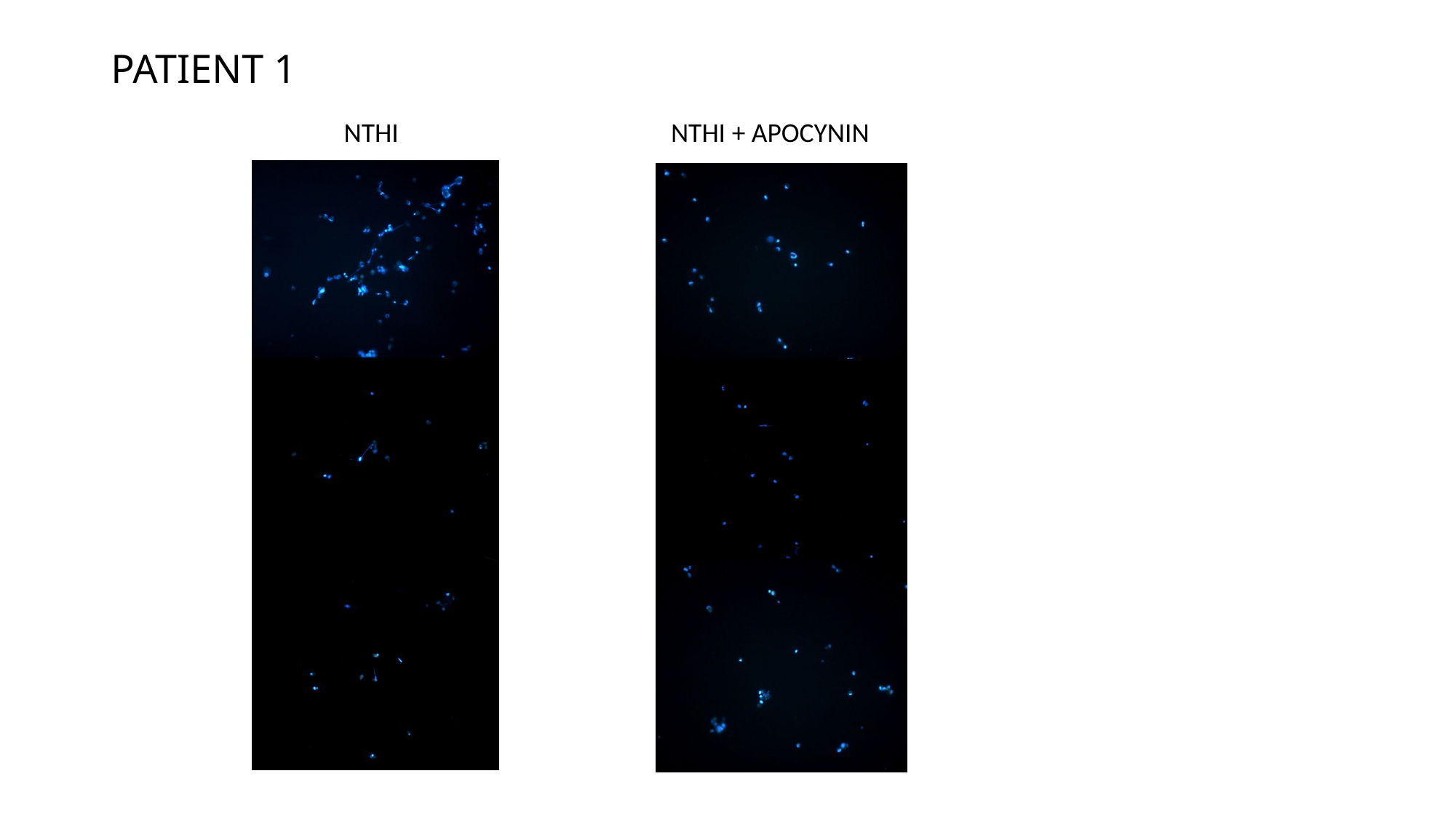

# PATIENT 1
NTHI
NTHI + APOCYNIN

## Slide 3
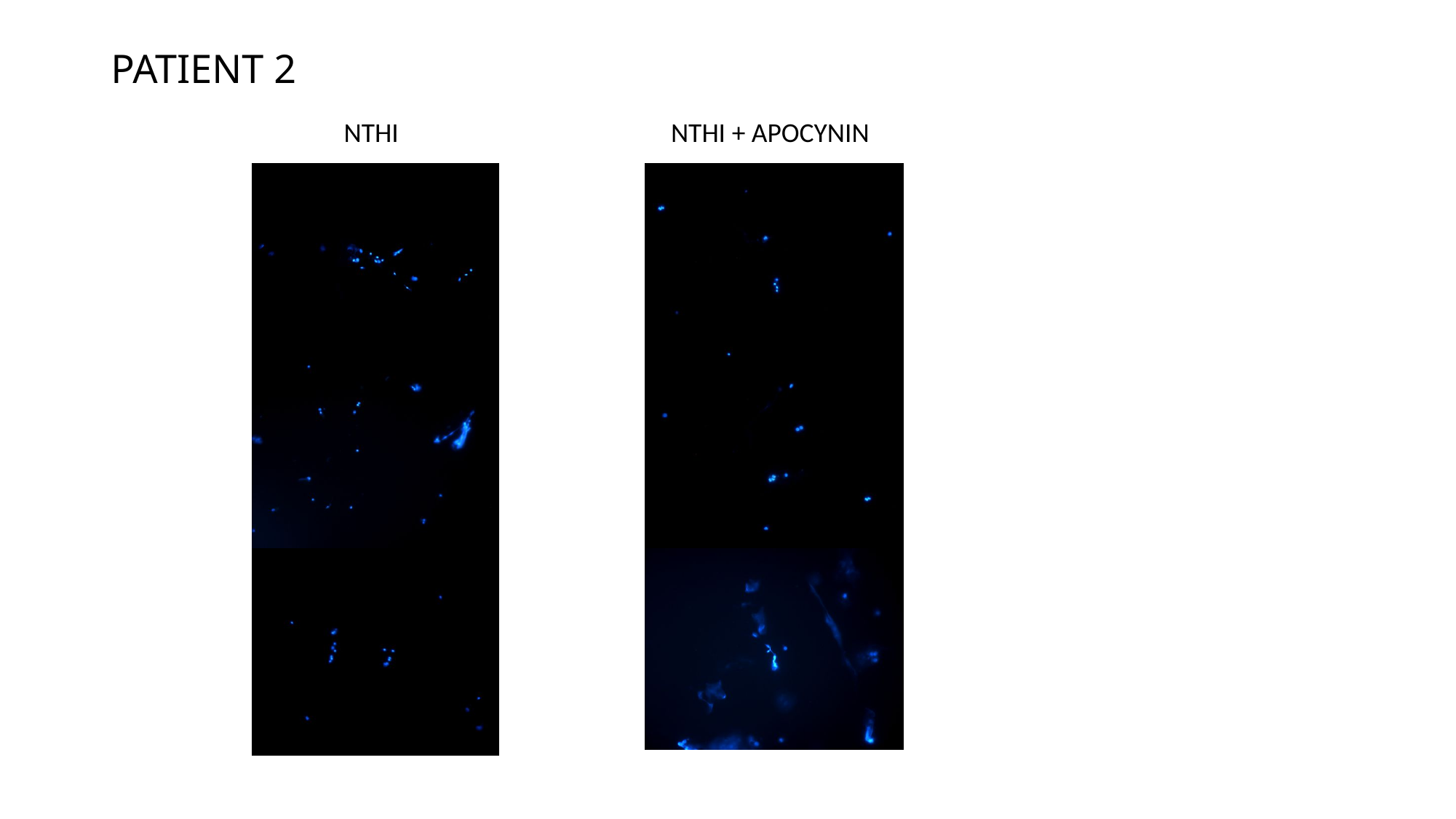

# PATIENT 2
NTHI
NTHI + APOCYNIN

## Slide 4
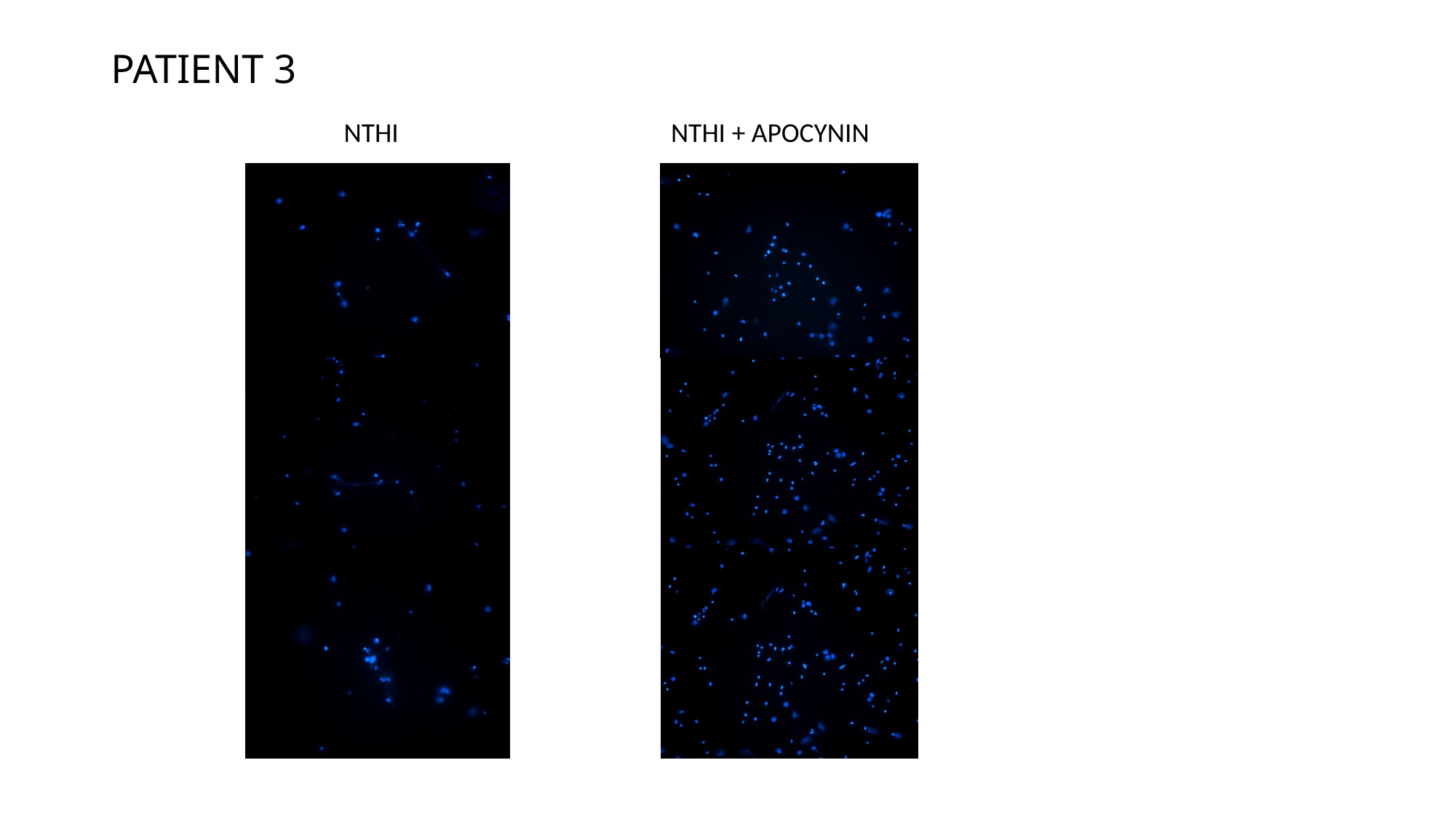

# PATIENT 3
NTHI
NTHI + APOCYNIN

## Slide 5
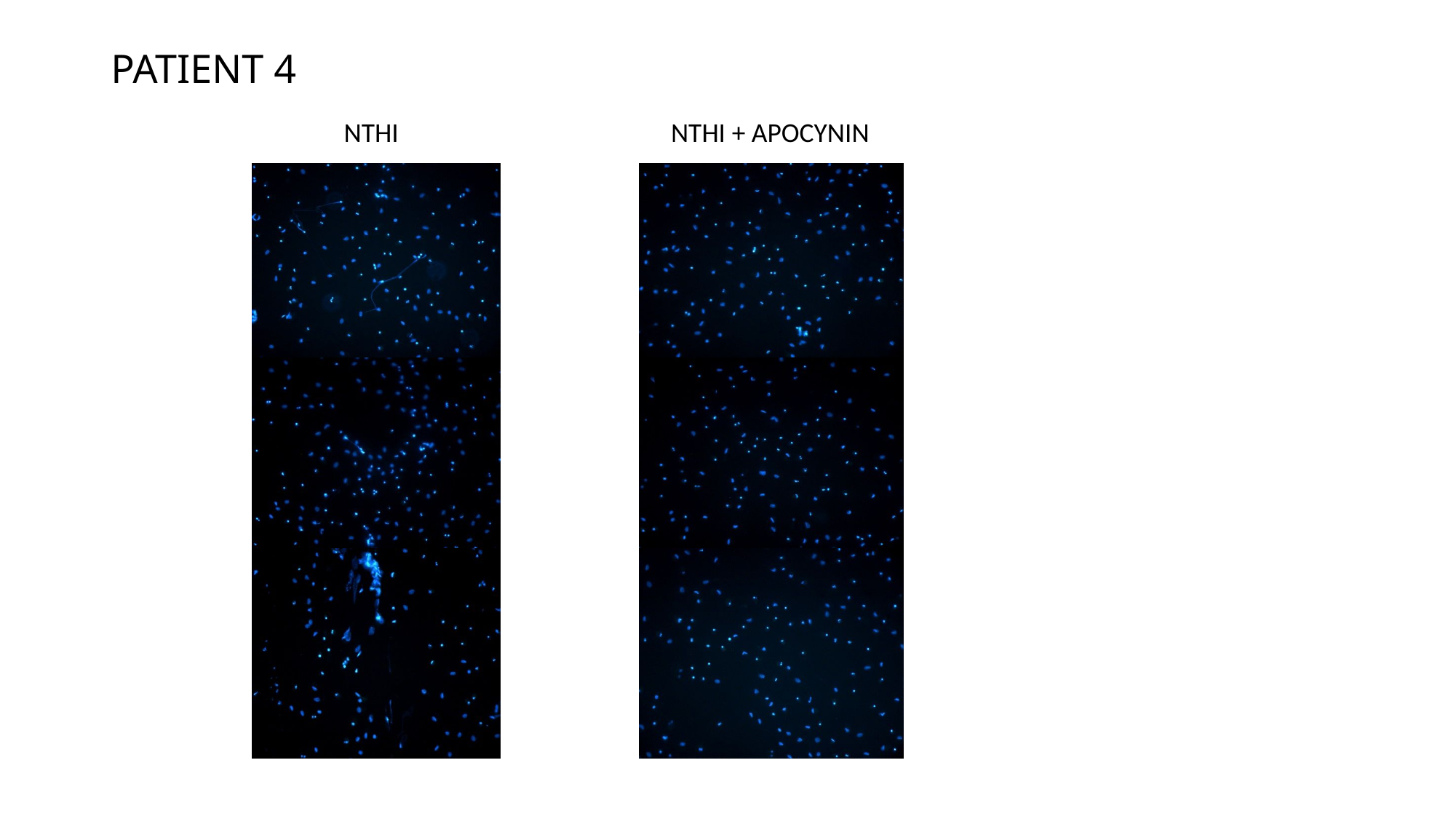

# PATIENT 4
NTHI
NTHI + APOCYNIN

## Slide 6
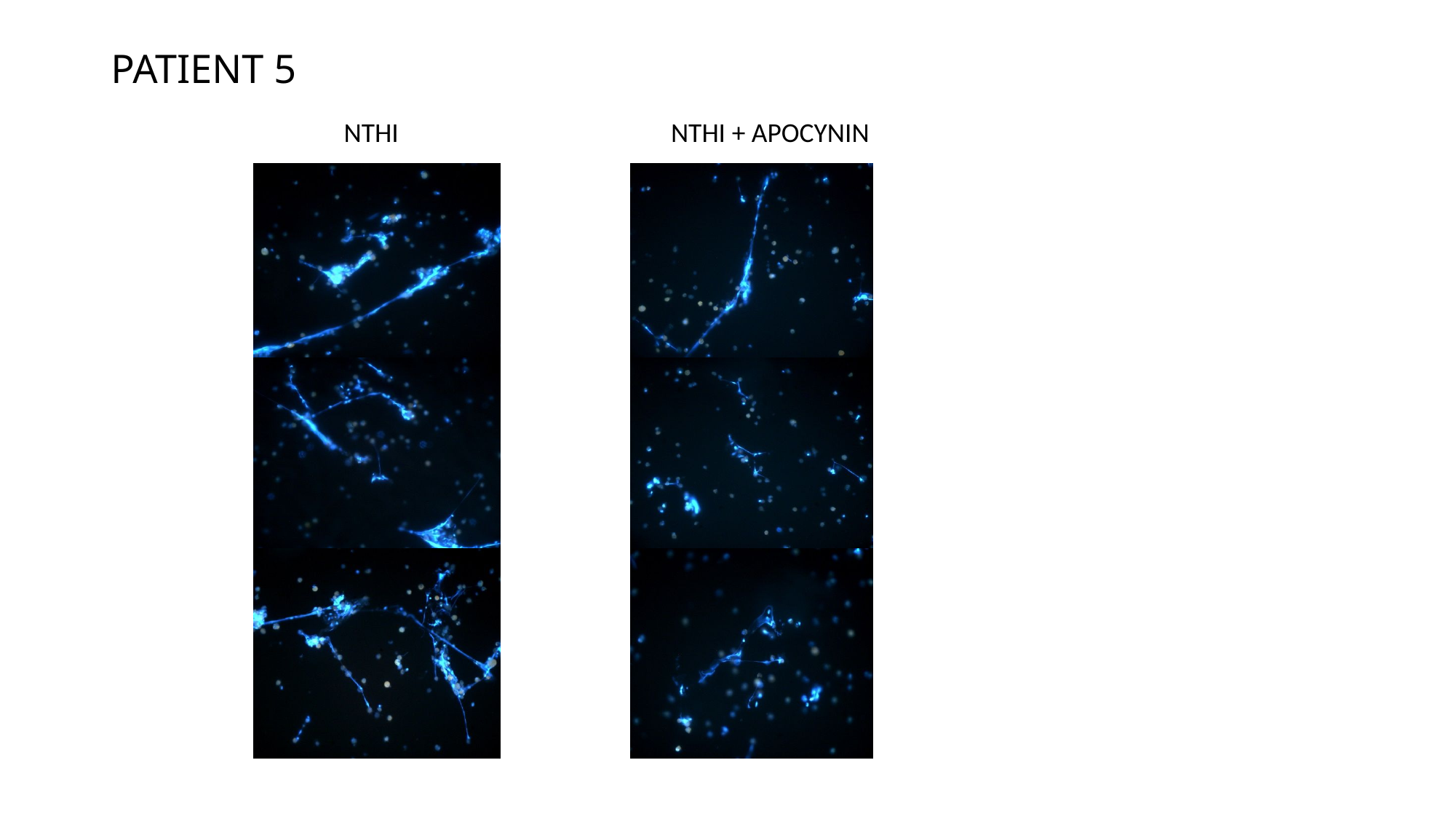

# PATIENT 5
NTHI
NTHI + APOCYNIN

## Slide 7
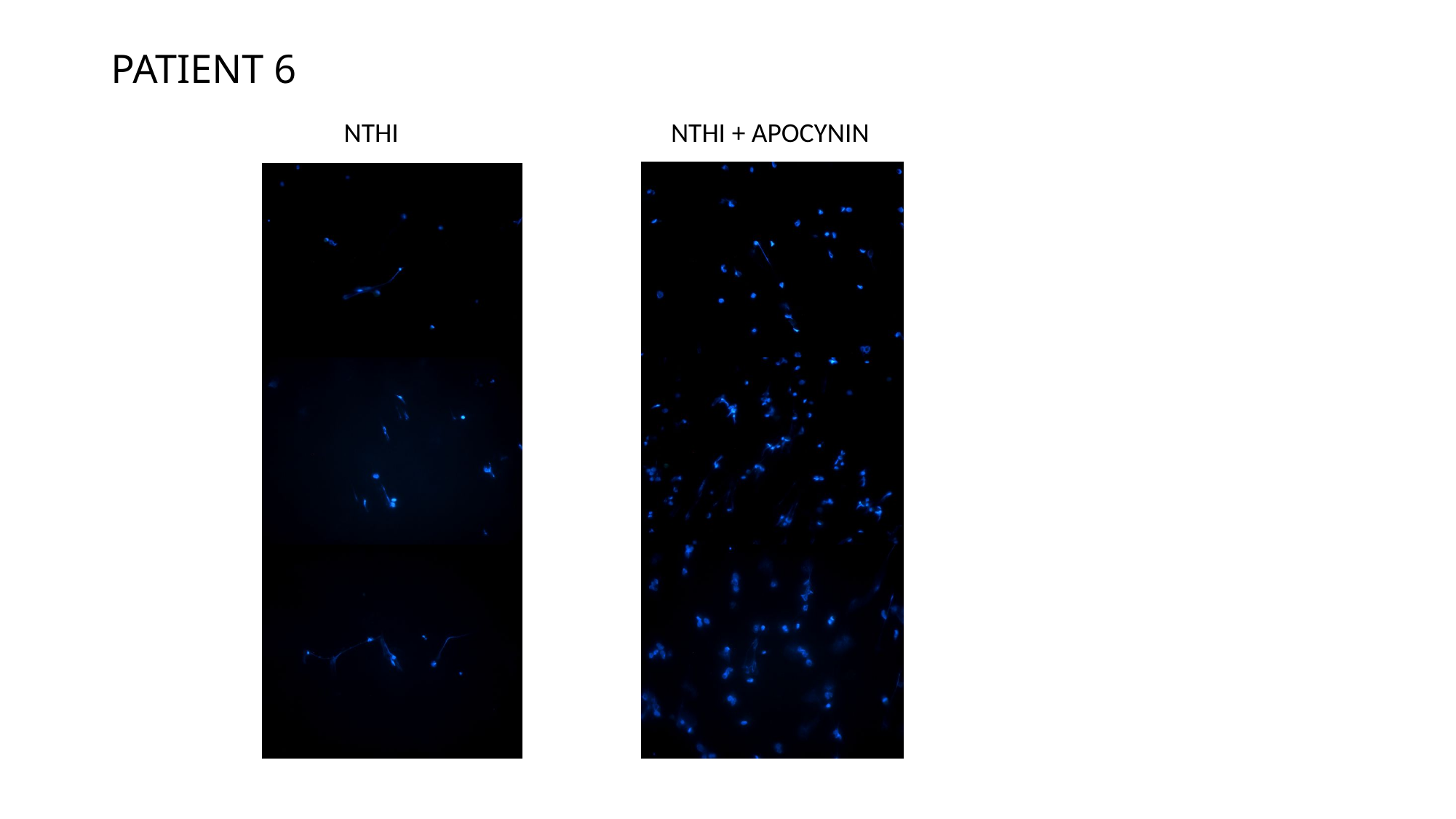

# PATIENT 6
NTHI
NTHI + APOCYNIN

## Slide 8
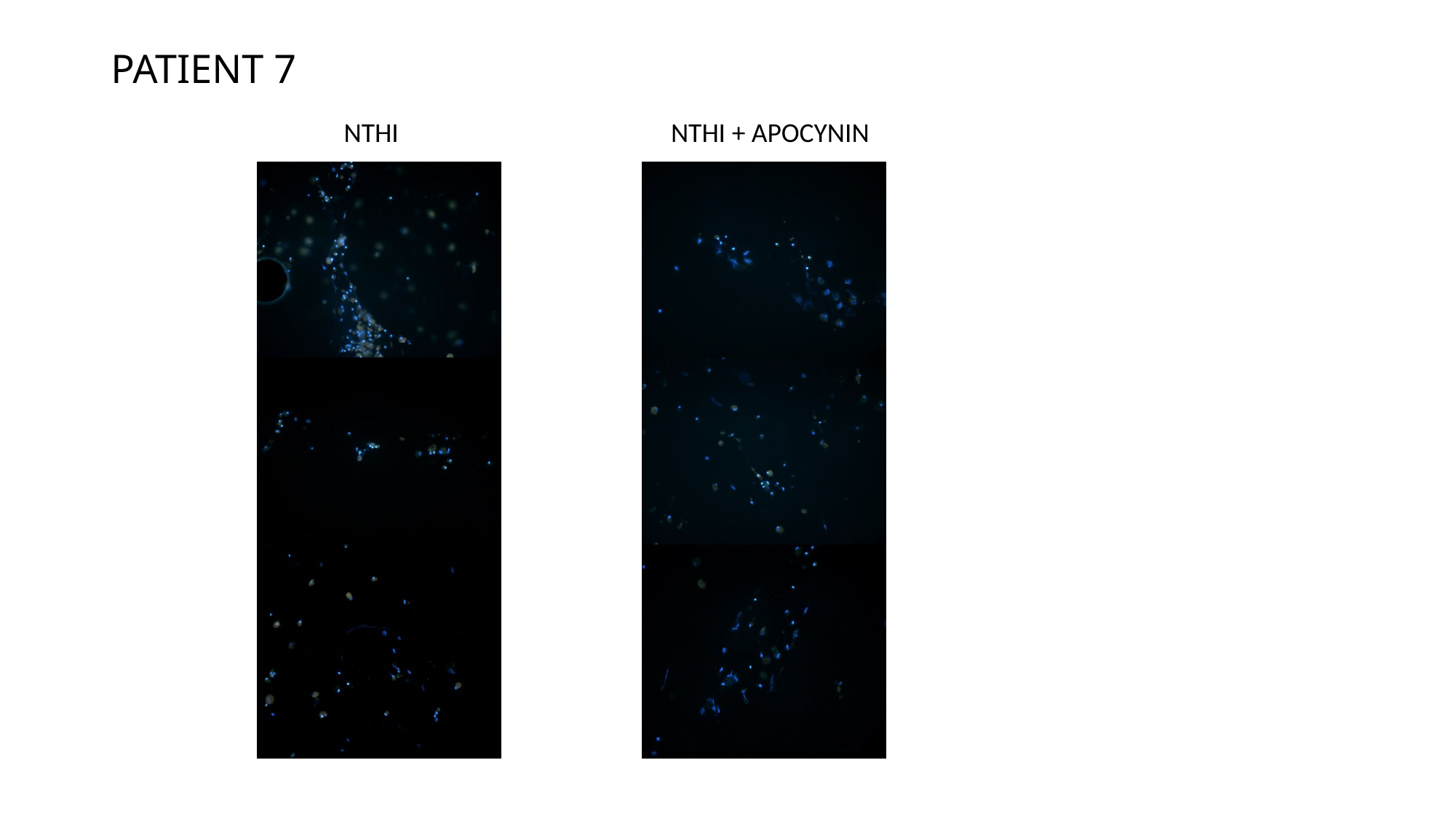

# PATIENT 7
NTHI
NTHI + APOCYNIN
