# Supplementary figures and images for: Nontypeable Haemophilus influenzae Induces Sustained Lung Oxidative Stress and Protease Expression
Source: PLoS One. 2015 Mar 20;10(3):e0120371. doi: 10.1371/journal.pone.0120371 (PMC4368769; doi:10.1371/journal.pone.0120371)

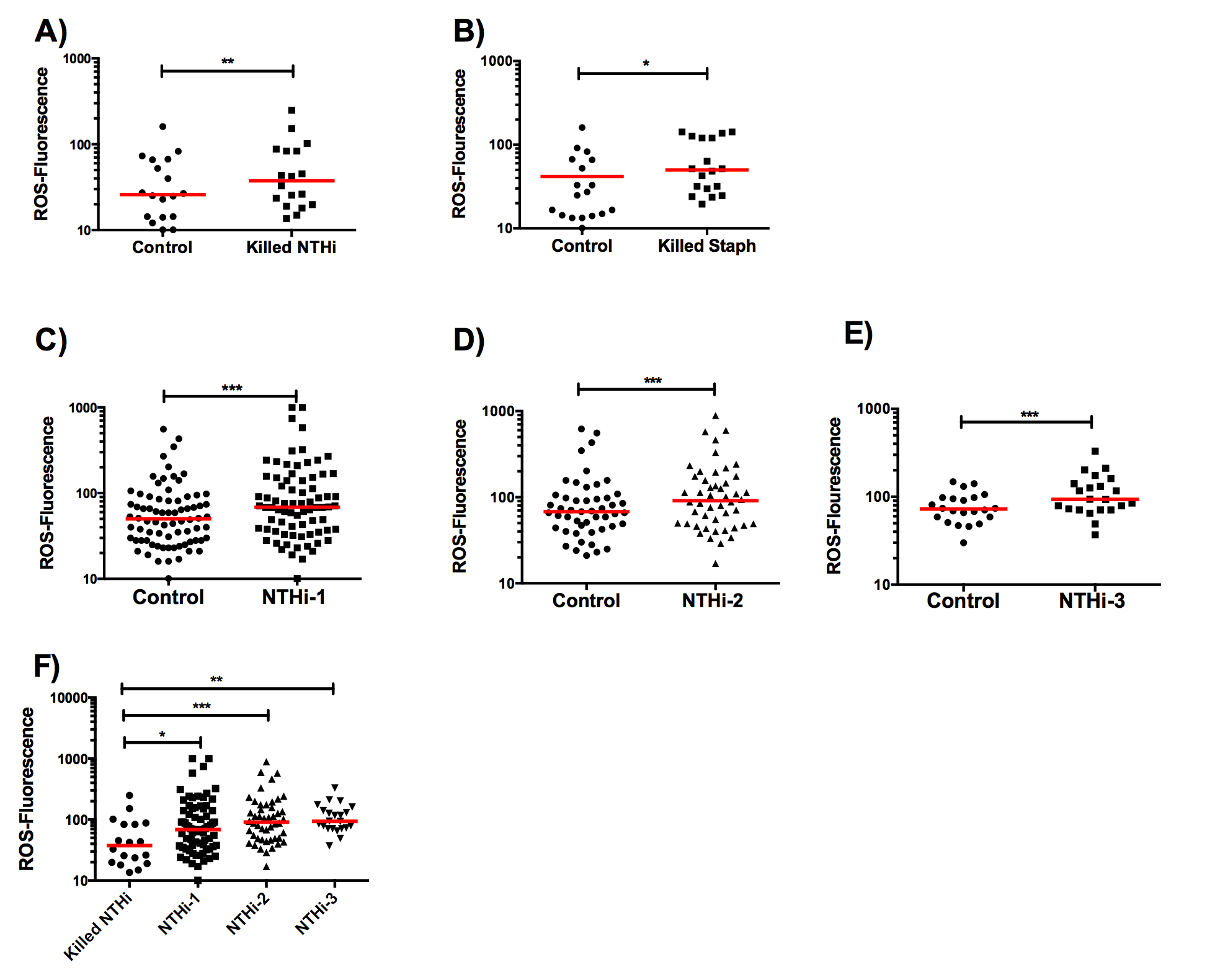

Supplement: S1 Fig — Response to killed bacteria: A) ROS production by lung phagocytes (n = 18 subjects) was significantly increased with killed NTHi antigen (control 20 (14–66), NTHi 37 (20–85)), (p = 0·002) (Wilcoxon matched-pairs rank test). B) ROS production by lung phagocytes (n = 18 subjects) was significantly increased by killed S. aureus antigen (control 26 (14–65), S. aureus 50 (29–122)), (p = 0·014) (Wilcoxon matched-pairs rank test). ROS response was similar with both bacteria. Lung phagocytes obtained from BAL were incubated with three different strains of NTHi (NTHi 1–3): C), NTHi-1 (control 50 (28–64), NTHi 68 (36–151)) (76 subjects), D), NTH-2 (control 68 (45–102), NTHi 91 (49–170)) (48 subjects) and E), NTH-3 (control 72 (57–98), NTHi 94 (71–140)) (22 subjects) all induced a significant increase in ROS (p<0·001) (Wilcoxon matched-pairs rank test). ROS production to killed NTHi (37 (20–85)), 18 subjects) was compared to live strains of NTHi-1 (68 (36–151)), 76 subjects), NTHi-2 (91 (49–170)), 48 subjects) and NTHi-3 (94 (71–140)), 22 subjects). Kruskall Wallis testing demonstrated overall significant difference (p = 0·002) and also significant increases when each live strain was compared to killed NTHi (For the phagocyte/macrophage assays, cells were obtained freshly from BAL and then put on rotation which would enhance baseline activity state). (TIF) [file pone.0120371.s010.tif]

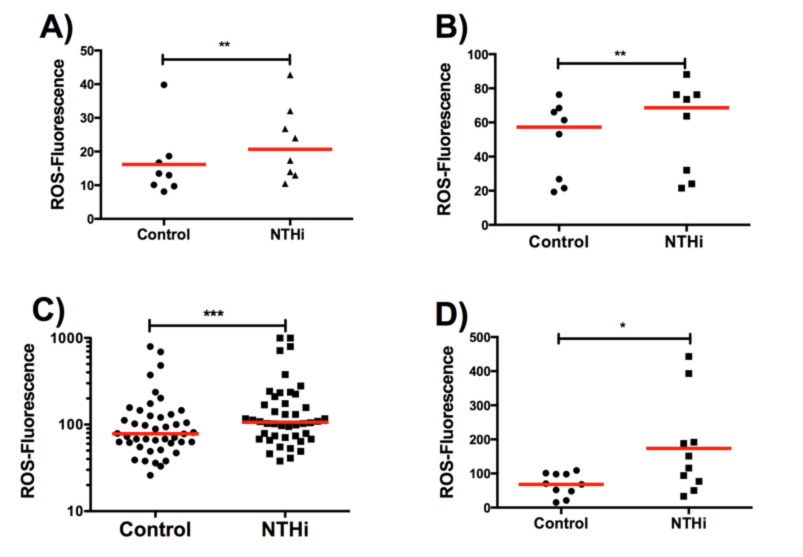

Supplement: S2 Fig — Cells types were stimulated with NTHi strain 1 and ROS measured using flow cytometry. A) Human fibroblast cell-lines (n = 8) had increased ROS (p = 0·008) following NTHi infection (control 13 (10–18), NTHi 21 (13–31)). B) Human epithelial cell-lines (n = 8) had increased ROS (p = 0·008) following NTHi infection (control 57 (23–68), NTHi 21 (13–31)). C) Human BAL macrophages (n = 44) had increased ROS (P< 0·001) following NTHi infection (control 80 (61–142), NTHi 107 (74–200)). D) Human blood neutrophils (n = 10) had increased ROS (p = 0·024) following NTHi infection (control 69 (41–99), NTHi 133 (70–242)). Analysis done by Wilcoxon matched-pairs rank test. (TIF) [file pone.0120371.s011.tif]

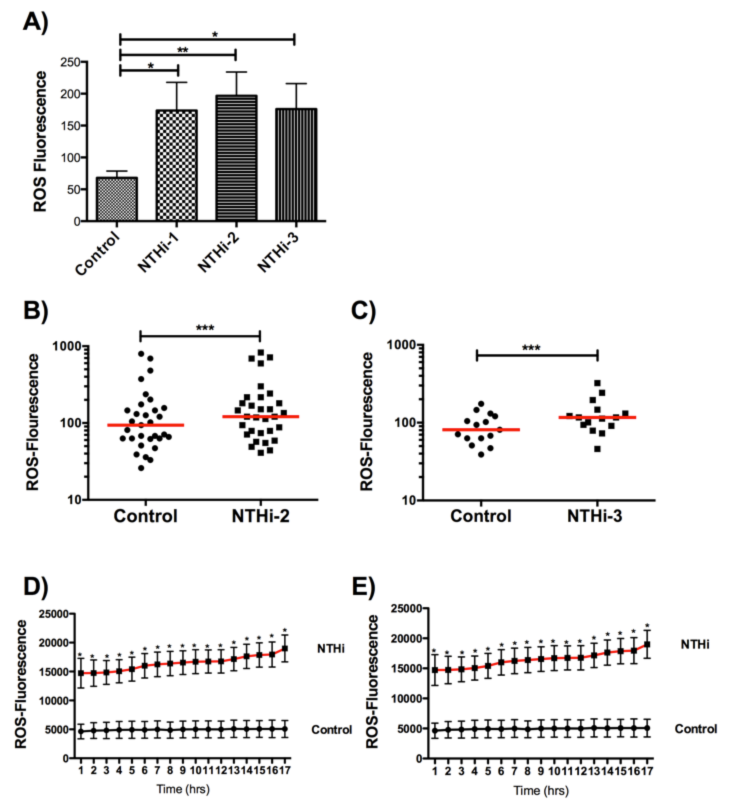

Supplement: S3 Fig — Neutrophils and macrophages were stimulated with multiple strains of NTHi to measure response. A) Peripheral blood from ten healthy controls was stimulated with three strains of live NTHi. Neutrophils were gated on, based on forward and side scatter and low levels of CD14 staining. There was a significant change in ROS production as measured by ANOVA, (p = 0·0008) with significant increases in each of the three strains compared to control (paired t-test) (control 68±11, NTHi-1 174±44, NTHi-2 197±37. NTHi-3 176±40). Lung macrophages obtained from BAL were incubated with two other strains of NTHI (NTH1–2 and NTHi-3): B), NTHi-2 (control 94 (63–157), NTHi-2 121 (74–217)) (31 subjects) and C), NTHi-3 (control 81 (63–121), NTHi-3 117 (91–147)) (15 subjects). All induced a significant increase in ROS production (p<0·001) (Wilcoxon matched-pairs rank test). Fluorescent microscopy was used to measure production of ROS over a 17-hour period in adherent macrophages, with readings of ROS-fluorescence taken every hour. B) Macrophages were stimulated with NTHi-2 and at all times there was a significant increase in ROS compared to control (p<0·001) (n = 15). C) Macrophages were stimulated with NTHi-3 and at all times there was a significant increase in ROS compared to control (p<0·01) (n = 11). All analyses done by paired t-testing. Levels of ROS were three-fold higher with NTHi stimulation (above control) with this method; compared to the approximately 40% increase when using the flow cytometry method (this difference is likely to be due to the cells for flow cytometry method being recently obtained from BAL (i.e. same day) and then being put on rotation and thus increasing the baseline activity state; when compared with the confocal assay where cells were seeded onto plates and allowed to rest overnight before being stimulated). (TIF) [file pone.0120371.s012.tif]

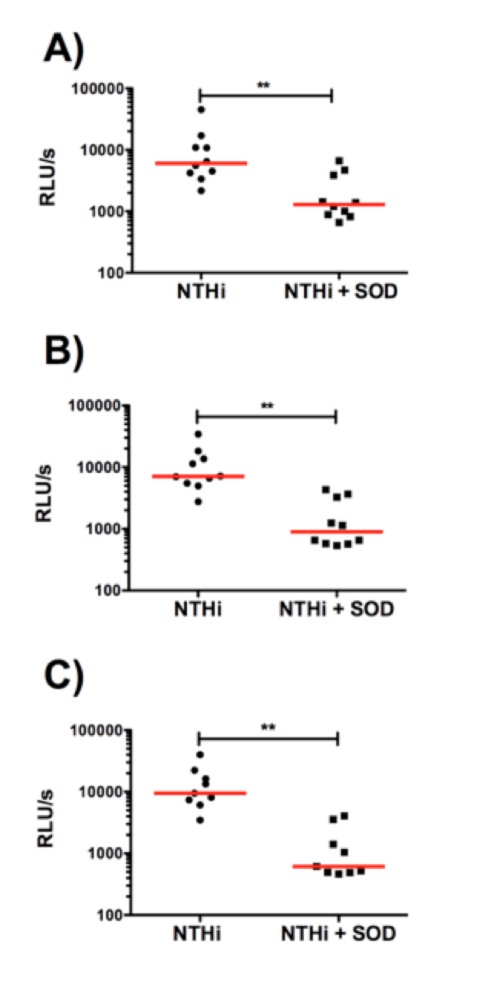

Supplement: S4 Fig — Adherent macrophages from ten subjects were stimulated with NTHi or NTHi with SOD. With all three strains there was significantly reduced ROS (expressed as RLU/s) A), NTHi-1 (NTHi 6037 (3990–12 461), NTHi & SOD 1291 (869–4086)), p = 0·004 B), NTHi-2 (NTHi 7101 (5364–14 824), NTHi & SOD 891 (574–3357)), p = 0·002 and C) NTHI-3, (NTHi 9446 (6750–19 323), NTHi & SOD 616 (491–2484)), p = 0·004 (Wilcoxon matched-pairs rank test). (TIF) [file pone.0120371.s013.tif]

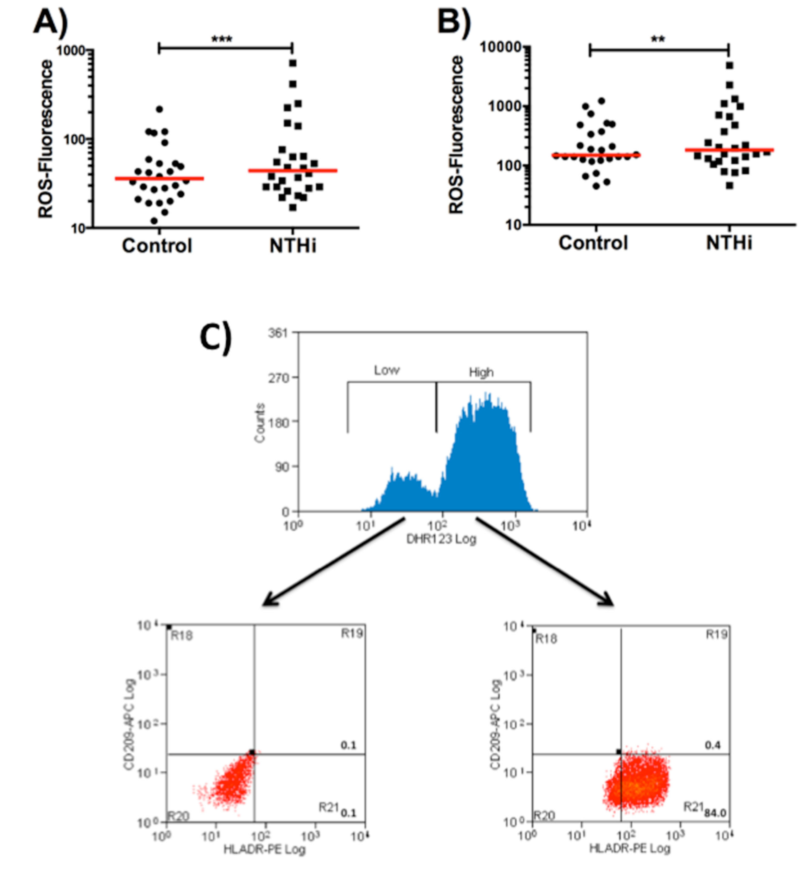

Supplement: S5 Fig — At baseline there were low and high-ROS producing populations of macophages (n = 26 subjects). Panel A demonstrates a significant increase in ROS production after NTHi stimulation in comparison to control (non-infected population) in the low-peak group (control 36 (21–55), NTHi 44 (27–92)), (p < 0·001). Panel B demonstrates a significant increase in ROS production after NTHi stimulation in comparison to control (non-infected population) in the high-peak group (control 143 (121–401), NTHi 163 (115–678)), (p = 0·004). Panel C shows the high-peak had a higher percentage of macrophages that express HLA-DR. (TIF) [file pone.0120371.s014.tif]

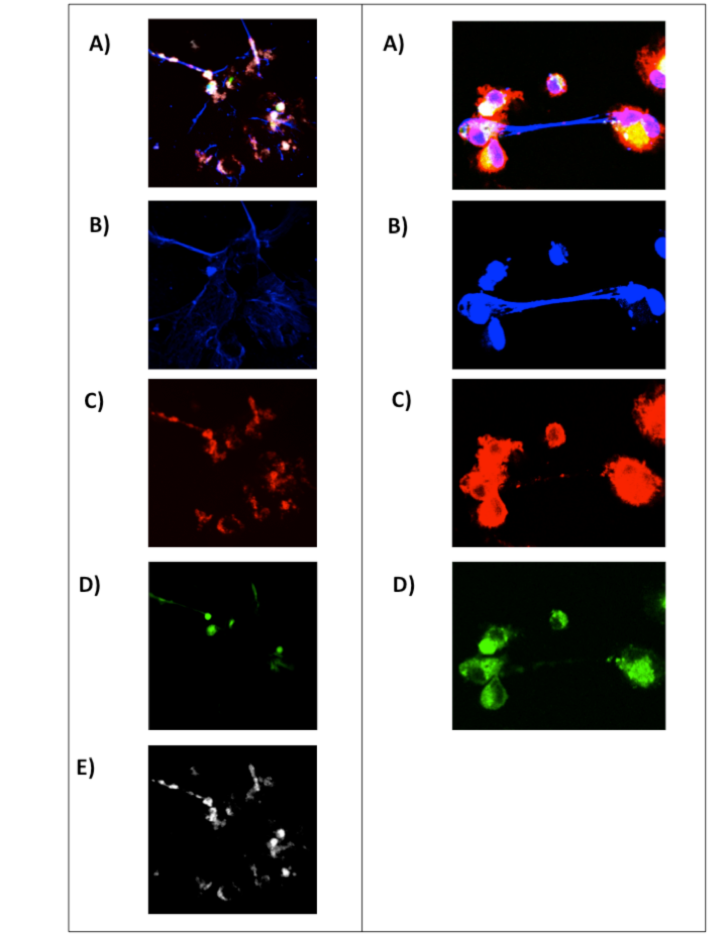

Supplement: S6 Fig — The left-hand column shows NET expression. Panel A) shows merged picture with Panel B) showing staining for chromatin, Panel C) shows staining for neutrophil elastase, Panel D) shows staining for ROS, and Panel E) shows staining for histone. The right-hand column shows MET expression. Panel A) shows merged picture with Panel B) showing staining for chromatin, Panel C) shows staining for macrophage metalloproteinase-12 and Panel D) shows staining for ROS. (TIF) [file pone.0120371.s015.tif]

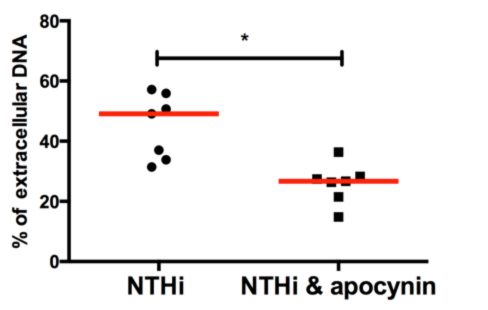

Supplement: S7 Fig — Macrophages were incubated with NTHi and apocynin was added to inhibit ROS production and the expression of METs as measured by the proportion of extracellular DNA of macrophages. Apocynin significantly decreased MET expression (NTHi 49 (34–56), NTHi & apocynin 27 (21–28)) (n = 7, p = 0·03) (Wilcoxon matched-pairs rank test). (TIF) [file pone.0120371.s016.tif]

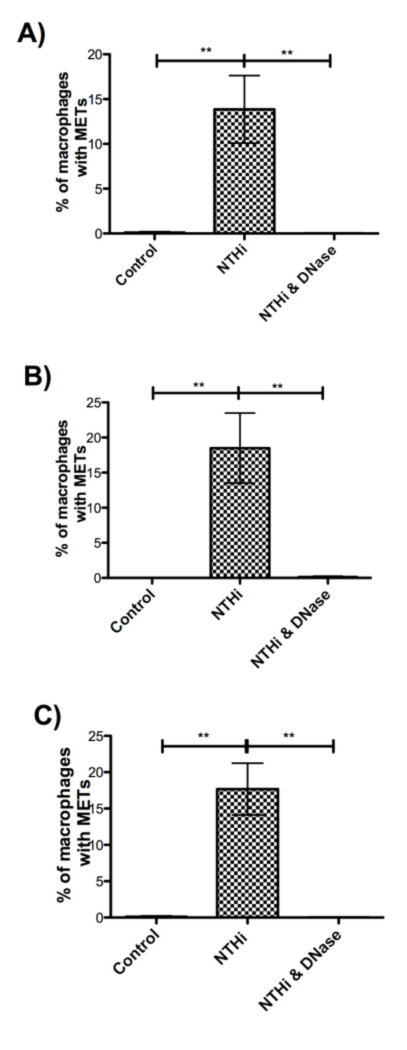

Supplement: S8 Fig — BAL macrophages (n = 8 subjects) were stimulated with NTHi and with/without DNase and the expression of METs measured by confocal microscopy at A), 20 minutes (control 0, NTHi 19± 5, NTHi & DNase 0.1±0.1) B), one hour (control 0, NTHi 19±5, NTHi & DNase 0.1±0.1) and C), three hours (control 0, NTHi 19±5, NTHi & DNase 0.1±0.1). At all 3 time-points there were significant differences (p<0·01) with both NTHi and DNase (paired t-testing). (TIF) [file pone.0120371.s017.tif]

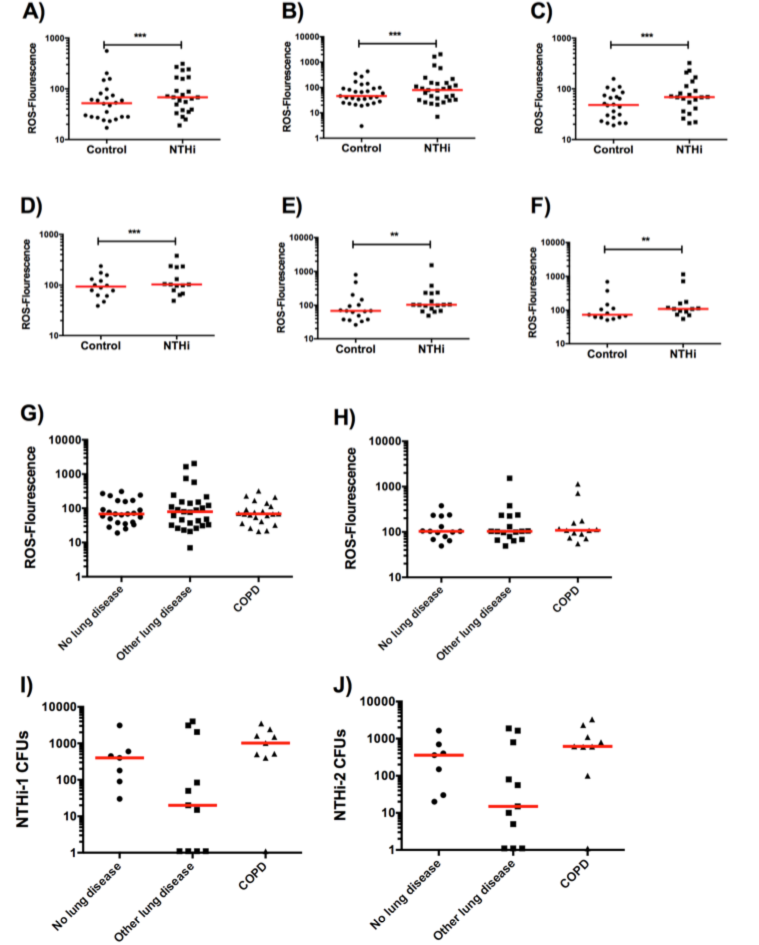

Supplement: S9 Fig — NTHi stimulation significantly increased ROS production to NTHi in lung phagocytes (A-C) and lung macrophages (D-F) in the three subgroups: A) No definable lung disease group (control 52 (28–99), NTHi 68 (38–165)), (n = 24 subjects), p<0·001, B), other lung disease group (control 46 (27–95), NTHi 79 (33–154)), (n = 29 subjects) p<0·001 and C), COPD group (control 48 (26–77), NTHi 69 (40–119)), (n = 22 subjects) p<0·001. ROS production to NTHi in lung macrophages: D) No definable lung disease group (control 93 (62–137), NTHi 103 (76–227)), (n = 14 subjects), p<0·001, E), other lung disease group (control 68 (38–124), NTHi 103 (74–229)), (n = 17 subjects) p<0·001 and F), COPD group (control 73 (61–129), NTHi 109 (82–166)), (n = 13 subjects) p = 0·008. All analyses by Wilcoxon matched-pairs rank test. G) There were no significant differences in the levels of ROS production by lung phagocytes stimulated with NTHi (p = 0·95). H) There were no significant differences in the levels of ROS production by lung macrophages stimulated with NTHi (p = 0·91). I) There were no significant differences in the bacterial killing of NTHi-1 in the patient groups (no definable lung disease group 410 (60–610)), other lung disease group 20 (0–2050), COPD group 1020 (450–2015)) (p = 0·18). J) There were no significant differences in the bacterial killing of NTHi-2 in the patient groups (no definable lung disease group 360 (30–700)), other lung disease group 15 (0–810), COPD group 620 (350–1710)) (p = 0·14). Statistical analysis performed with one-way ANOVA and Kruskal-Wallis test. (TIF) [file pone.0120371.s018.tif]
