# Supplementary material for: Nontypeable Haemophilus influenzae Induces Sustained Lung Oxidative Stress and Protease Expression
Source: PLoS One. 2015 Mar 20;10(3):e0120371. doi: 10.1371/journal.pone.0120371 (PMC4368769; doi:10.1371/journal.pone.0120371)
Supplement: S2 Table — ROS is the measure of fluorescence induced by DHR-cleavage with results expressed as median and interquartile ranges. Statistical analysis performed using Wilcoxon matched-pairs rank test. (PDF) [file pone.0120371.s020.pdf]

**Bacterial strain**	**Number of subjects**	**ROS Control**	**ROS Bacteria added**	***p***
NTHi-1	44	80<br>61-142	107<br>74-200	< 0·001
NTHi-2	31	94<br>63-157	121<br>74-217	<0·001
NTHi-3	15	81<br>63-121	117<br>91-167	<0·001
