# Supplementary material for: Nontypeable Haemophilus influenzae Induces Sustained Lung Oxidative Stress and Protease Expression
Source: PLoS One. 2015 Mar 20;10(3):e0120371. doi: 10.1371/journal.pone.0120371 (PMC4368769; doi:10.1371/journal.pone.0120371)
Supplement: S3 Table — Chemiluminescence is expressed as reactive light units per second (RLU/s). Statistical analysis performed by one-way analysis of the variance using Friedman test. (PDF) [file pone.0120371.s021.pdf]

**N=15 subjects**	**Control**	**NTHi-1**	**NTHi-2**	**NTHi-3**	***p***
Chemiluminescence					
Median	3084	4502	6609	7392	
<0·001					
Interquartile range	1085-10050	1155-11201	1328-13681	1152-16274	
