# Supplementary material for: Nontypeable Haemophilus influenzae Induces Sustained Lung Oxidative Stress and Protease Expression
Source: PLoS One. 2015 Mar 20;10(3):e0120371. doi: 10.1371/journal.pone.0120371 (PMC4368769; doi:10.1371/journal.pone.0120371)
Supplement: S4 Table — The % of neutrophils or macrophages expressing extracellular traps (either as NETs or MET-like structures) as control, after NTHi stimulation and with the combined effect of NTHi and DNase (different timepoints of 20 minutes, 1 hour and 3 hours) (PDF) [file pone.0120371.s022.pdf]

NET formation at 1 hour

Control	NTHI	NTHi & DNase
0	50	0
8.3	60.5	1.8
10	41.7	4.8
0	45.8	17
7.7	26.7	7.1
4.8	15.2	0
15	40.9	

MET formation at 20 minutes

Control	NTHI	NTHi & DNase
0.8	8.3	0
0	7.6	0
0	16.6	0
0	7	0
0	33.3	0.3
0	4.6	0
0	26.5	0
0	7	0

MET formation at 1 hour

Control	NTHI	NTHi & DNase
0	6.45	0
0	10.6	1.07
0	21.2	0
0	4.59	0
0	27.9	0
0	8	0
0	23.07	0
0	46.15	0

MET formation at 3 hours

Control	NTHI	NTHi & DNase
0	18.8	0
0	15.55	0
0	14.2	0
0.99	11.57	0.26
0	40	0
0	5.26	0
0	19.4	0
0	16.6	0
