# Supplementary material for: Nontypeable Haemophilus influenzae Induces Sustained Lung Oxidative Stress and Protease Expression
Source: PLoS One. 2015 Mar 20;10(3):e0120371. doi: 10.1371/journal.pone.0120371 (PMC4368769; doi:10.1371/journal.pone.0120371)
Supplement: S1 Text — (DOCX) [file pone.0120371.s023.docx]

**Supporting information**

Nontypeable *Haemophilus influenzae* induces sustained lung oxidative stress and protease expression

Paul T King,^1, 2^ Roleen Sharma,^1, 2^ Kim O’Sullivan,^1^ Stavros Selemidis,^3^ Steven Lim,^1^ Naghmeh Radhakrishna,^2^ Camden Lo,^4^ Jyotika Prasad,^2^ Judy Callaghan,^4^ Peter McLaughlin,^2^ Michael Farmer,^2^ Daniel Steinfort,^2^ Barton Jennings,^2^ James Ngui,^5^ Bradley RS Broughton,^3^ Belinda Thomas,^2, 6^ Ama-Tawiah Essilfie,^7^ Michael Hickey,^1^ Peter W Holmes^2^ Philip Hansbro,^7^ Philip G Bardin,^2, 6^ and Stephen R Holdsworth^1^

1. Monash University Department of Medicine/Monash Medical Centre, Melbourne, Australia
2. Monash Lung and Sleep, Monash Medical Centre
3. Department of Pharmacology, Monash University
4. Monash Micro Imaging, Monash University
5. Clinical Immunology, Monash Medical Centre
6. Monash Institute of Medical Research
7. School of Biomedical Sciences and Pharmacy, University of Newcastle, Newcastle, Australia

**TABLE OF CONTENTS**

**SECTION 1: ADDITIONAL METHODOLOGICAL INFORMATION**

- 1. Bronchoalveolar lavage (BAL) patients
  2. Bronchoscopy
  3. Processing of the BAL
  4. Cell lines
  5. Neutrophils
  6. Bacteria
  7. Flow cytometry
  8. Fluorescent microscopy
  9. Killing assay
  10. Chemiluminescence
  11. In vivo production of reactive oxygen species (ROS) in mouse lungs
  12. Statistical analysis

**SECTION 2: REFERENCES**

**SECTION 1: ADDITIONAL METHODOLOGICAL INFORMATION**

**1.1 Bronchoalveolar lavage (BAL) patients**

Patients were referred in to have a bronchoscopy at Monash Medical Centre (MMC)/Monash Health. Of the 121 patients, 116 of the subjects were referred in after assessment by an affiliated respiratory physician; whilst five other subjects were referred by other specialist physicians. Subjects had lung function testing performed using GOLD guidelines (Forced expiratory volume in one second/forced vital capacity ratio <0.7 and not fully reversible) [[1](#_ENREF_1)] with the best post-bronchodilator response used for results. Subjects also had computed tomography (CT) scans, which were reported by a specialist radiologist. Forty subjects of the total cohort had a significant smoking history (> than 10 pack-years) and most of these subjects (29 in total) had chronic obstructive pulmonary disease (COPD). Only four subjects of the total group were active smokers at the time of bronchoscopy. Three of the subjects were inpatients and the rest were outpatients.

Nontypeable *Haemophilus influenzae* (NTHi) is the most common bacterium isolated in subjects with COPD. However it is also a major bacterium associated with a number of other inflammatory respiratory conditions including pneumonia, bronchitis, bronchiectasis/cystic fibrosis, otitis media and sinusitis [[2](#_ENREF_2)]. In addition it has also been shown to extensively invade the lung in a variety of end-stage pulmonary conditions including COPD, cystic fibrosis and interstitial lung disease [[3](#_ENREF_3),[4](#_ENREF_4)]. Therefore this bacterium may have a potentially important role in a wide variety of lung conditions in addition to COPD.

To further categorize the immune response to NTHi we divided the subjects into three subgroups. On the basis of the physician assessment, lung function testing, CT scanning and results from bronchoscopy the 121 patients were divided into three groups; 1), those with no definable cause for their respiratory symptoms, (n=34) 2), subjects with COPD (n=40) and 3), those with inflammatory lung disease but without COPD (n=47). Characteristics of the three different groups are listed in table 1 (in main text). The subjects with no definable lung disease, acted as a negative control group. We chose the other group who had lung inflammation but no COPD as another group to contrast to that with the COPD subjects.

The subjects with inflammatory lung disease but without COPD had either sarcoidosis (21 subjects), infective lung disease (eleven subjects had bronchiectasis and eleven subjects had current or recent pneumonia), two had asthma, and two had interstitial lung disease. The indications for bronchoscopy were to diagnose sarcoidosis or for those with infective lung disease/other lung disease to assist with microbiologic diagnosis/profile of airway inflammation. In this cohort, four subjects were being currently treated with systemic immunosuppressive medication; one renal transplant subject (tacrolimus and prednisolone), two patients with ulcerative colitis (prednisolone) and one patient with Crohn’s disease and systemic lupus erythematosis (azathioprine and infliximab). In this cohort, seven subjects were documented in the file to have used a macrolide antibiotic in the past month including one who had been treated with a long-term regimen for mycobaterium avium complex. In this cohort, eight subjects were documented to have had a significant smoking history. In this group, five subjects had *H. influenzae* isolated from the bronchoscopy. In the pneumonia patients, ten out of eleven subjects had had a clinical diagnosis of respiratory infection in the past 5 weeks that had been treated with antibiotics and all of the bronchiectasis patients had symptoms of recurrent airway infection and had received antibiotics in the past year. Eight subjects had a significant smoking history (≥ 10 pack years).

The primary indications for performing the bronchoscopies on the subjects in the COPD group were for assessment of chronic cough (eleven subjects), exclusion of malignancy (ten subjects), airway infection (ten subjects), assessment of possible interstitial lung (five subjects) disease, haemoptysis (three subjects) and tracheobronchomalacia (one subject). The subjects in the COPD group had a much higher use of inhalational corticosteroids (32/40 or 80%, with a median dose of 400 mcg of budesonide equivalent) compared to those without definable lung disease (4/34 or 12%) and the group with other lung disease (21/47 or 45%). Of the COPD subjects, five of them had other active lung disease; seven had lung cancer, one had sarcoid and two had asthma. In this cohort, two subjects were currently being treated with systemic immunosuppressive medication; one subject with rheumatoid arthritis (methotrexate and prednisolone) and one subject who was receiving chemotherapy for lymphoma. In this cohort, six subjects were documented in the file to have used a macrolide antibiotic in the past month. In this cohort, thirty one subjects were documented to have had a significant smoking history. In this group, five subjects had *H. influenzae* isolated from the bronchoscopy.

Of the 34 patients with no definable lung disease, the indications for bronchoscopy were; chronic cough in 23 subjects, hemoptysis in 9 subjects and lung nodule in two patients. In this cohort, two subjects were currently being treated with systemic immunosuppressive medication; one subject with monoclonal gammopathy of undertermined significance (prednisolone) and one subject who was receiving chemotherapy for renal carcinoma. In this cohort, four subjects were documented in the file to have used a macrolide antibiotic in the past month. In this cohort, three subjects were documented to have had a significant smoking history. In this group, two subjects had *H. influenzae* isolated from the bronchoscopy.

Consent to obtain a BAL sample was obtained prior to bronchoscopy.

**1.2 Bronchoscopy**

Bronchoscopy was performed in the bronchoscopy suite at MMC/Monash Health. BAL was performed as previously described [[5](#_ENREF_5)] with multiple warmed aliquots of 25 ml of normal saline injected into the right middle lobe and aspirated.

**1.3 Processing of the BAL**

Approximately 10 ml of the returned fluid was sent to the Cytology Department in the Pathology Laboratories of Monash Health where differential counting for white cell subtypes was performed. The remainder of the BAL was taken to the laboratories of the Department of Medicine, MMC. Samples were then spun down and washed twice then re-suspended in culture media (RPMI-1640 (Sigma-Aldrich, St Louis, MO, USA), 10% fetal calf serum, 0.1% L-glutamine (Sigma-Aldrich) and antibiotics (1% Penicillin (Sigma-Aldrich), 1% Streptomycin (Sigma-Aldrich) and 180 μg/ml Metronidazole (Claris, Chachamadi, Ahmedabab, India)). Trypan blue exclusion was used to obtain a cell count. Ammonium choloride was used to lyse red blood cells. All subjects had > than 85% trypan blue exclusion with a mean viable macrophage count of 4.3 × 10^6^. For the flow cytometry assays, cells were put on rotation to prevent adherence of the macrophages, using a MACSmix tube rotator (Miltenyi Biotec, Bergish Gladbach, Germany). For chemiluminescence and confocal assays, macrophages were seeded onto 96-well flat-bottomed plates at a concentration of 25 000 cells per well; whilst for the killing assay cells were seeded onto 24-well flat-bottomed plates at concentration of 100 000 cells per well using a previously described method [[6](#_ENREF_6)].

**1.4 Cell lines**

Primary human bronchial epithelial cells were obtained during routine bronchoscopy and cultured from bronchial brushings on collagen-coated flasks (MP Biomedicals, Santa Ana, CA, USA) in supplemented bronchial epithelial growth medium (BEGM; Lonza, Australia). Primary human airway fibroblasts were obtained from resection or biopsy as described previously [[7](#_ENREF_7)] and cultured in minimum essential medium (MEM, Gibco, USA) supplemented with 10% FCS, 2 mM L-glutamine, 100 U/mL penicillin and 100 µg/mL streptomycin. Consent to use the cells to develop cell-lines was obtained prior to bronchoscopy. These cell lines were obtained from different subjects than the 121 who had BAL samples taken who are described in section 1.1.

For fibroblast cell lines, all patients were non-smokers or had not smoked for >15 years and none had a diagnosis of asthma or COPD (normal FEV_1_ measurements). Bronchial biopsies and brushings were obtained from the same anatomical regions (bronchial generations 4-7).

The same methods were used to obtain eight epithelial cell lines; five were from healthy controls and three from mild asthmatics.

**1.5 Neutrophils**

Peripheral blood neutrophils were obtained from authors and laboratory staff with consent prior to sample being taken. Neutrophils were isolated by density gradient using Polymorphprep (Progen Biotechnik, Heidelberg, Germany)**.**

**1.6 Bacteria**

Four previously described strains of NTHi were used MU/MMC 1-4 [[8](#_ENREF_8)]. Bacteria had been previously typed and sequenced and demonstrated to be NTHi. We also used a newly isolated strain of NTHi from a patient with severe COPD and recurrent exacerbations. This strain was characterized and sequenced as previously described and was found to be 99% homologous with sequences corresponding to *H. influenzae* (American Type Culture Collection 9795). This strain was designated as MU/MMC5. For the killed-antigen preparation NTHi strains MU/MMC 1-4 were heat-inactivated and fixed in 2% paraformaldehyde as previously described [[9](#_ENREF_9)] For the live strains we used MU/MMC 1 for most of the experiments, designated as NTHi-1 for this study (this was the main strain that we have used recently). As NTHi has significant variation we also used two other live strains to test responses, MU/MMC-5 designated as NTHi-2, and MU-MMC 2 designated as NTHi-3. The live bacteria were cultured up from frozen stock (kept in -80^0^C freezer) approximately every 3 months. Bacteria were grown at 37^0^C in 5%CO_2_ in an incubator on chocolate agar plates and nutrient-broth to logarithmic phase and numbers of bacteria were calculated by optical density with spectrometry as previously described [[8](#_ENREF_8)]. We also used a commercially available preparation of killed *Staphylococcus aureus* (Zysorbin, Life Technologies, Carlsbad, CA, USA) as a positive control to validate BAL cell response as previously described [[10](#_ENREF_10)].

**1.7 Flow cytometry**

We used a well-established flow cytometry method to measure the production of ROS from lung phagocytic cells [[10](#_ENREF_10),[11](#_ENREF_11)]. The production of ROS causes cleavage of the dye dihydrorhodamine 123 (DHR), (Life Technologies), which leads to an increase in intracellular fluorescence measured on a Legacy MoFlo cytometer (Beckman Coulter, Brea, CA, USA). Cells were washed and incubated in antibiotic-free media with bacteria and DHR (at 5 μg/mL) for an hour on rotation at 37^o^C. Both killed and live bacteria were added at multiplicity of infection (MOI) of 100:1. This MOI is a standard commonly used for NTHi infection assays. We used anti-CD14 APC/Cy7 (BD Biosciences, San Jose, CA, USA) to identify lung macrophages; HLA-DR (BD Biosciences) and CD209 (eBioscience, San Diego, CA, USA) were used to further characterize for M-1 and M-2 macrophages respectively. The surface antibodies were added post bacterial-inoculation and appropriate isotype and fluorescence with controls were used for analysis. Lung phagocytic cells (both macrophages and neutrophils) were gated on, based on forward and side-scatter using Summit software (Version 4.3; Beckman Coulter).

MMP12 staining Protocol

Control and NTHi-stimulated BAL macrophages were surface-stained with MMP12 (Novus Biologicals, Littleton, CO, USA) antibody for 60 min on ice. Cells were washed twice in 1 x PBS, incubated with Alexa Fluor 488 chicken anti-rabbit IgG antibody for 45 minutes on ice, then washed twice with 1 x phosphate buffered saline (PBS) and resuspended in PBS containing Propidium Iodide (Sigma Aldrich) before cells were analysed on BD FACSCantoII Flow Cytometer.

**1.8 Fluorescence microscopy**

We adopted the DHR flow cytometry method to measure the production of ROS over time. Cells were seeded onto 96-well flat-bottomed plates and washed to remove non-adherent cells. At least 24 hours prior to assays, wells were washed twice to remove antibiotics and incubated in antibiotic-free media. The three live strains of NTHi were stained with D-DIO fluorescent dye (Molecular Probes, Eugene, OR, USA) in solution for 20 minutes then washed twice and suspended in culture medium. Cells were then incubated with various strains of live NTHi at an MOI of 100:1 (bacteria to macrophages) and DHR added to each well at a concentration of 5 μg per ml. Plates were then mounted on a confocal microscope with a heated stage. Specimens were imaged live using a Nikon C1 confocal laser scanning microscope (Nikon, Tokyo, Japan) with a 10x 0.45NA lens, with an incubation chamber built around the system maintaining 37^o^C, 5% CO_2_ gas incubation conditions at all times. Hourly measures of DHR fluorescence as a measure of ROS were done over a 17-hour time period. DHR signal intensity were analyzed by Imaris v7.6 (Bitplane AG, Zurich, Switzerland) where cells were identified individually and DHR fluorescence tracked over time. For each outcome duplicate wells were measured with readings taken from 25 individual cells (i.e. total of 50 individual cells for each outcome). Plates were analyzed for 17 hours with readings of cells taken every hour. DHR is an intracellular dye and its presence a marker of intracellular ROS production. With the confocal microscopy we take multiple cross sectional pictures through the macrophages (more than ten sections) and in this way we can accurately co-localize the intracellular ROS with the presence of intracellular bacteria.

Confocal microscopy staining protocol

BAL macrophages (100,000 per sample) or peripheral blood neutrophils from healthy donors were seeded (400,000 per sample) out on poly-L-lysine coated coverslips and incubated with DHR to detect ROS production and fluorescently labelled NTHi (D-DIO fluorescent dye, Molecular Probes) at 20 minutes, 1 hour and 3 hours for macrophages, and 1 hour for neutrophils. DNase treated cells were incubated with DNase (Sigma-Aldrich) at 2 µg/ml for 10 minutes prior to addition of NTHi. Cells were fixed in 2% paraformaldahyde-periodate and lysine for 10 minutes, washed briefly in PBS, permeabilised in 0.2% Tween 20 in PBS for 20 minutes followed by blocking with 10% chicken sera in 5% bovine albumin sera (BSA) diluted in PBS for 30 minutes. Primary antibodies were incubated for 1 hour at RT at the following concentrations: Rabbit anti-human neutrophil elastase (Abcam, Cambridge, U.K.) 1:100, rabbit anti human MMP12 (Novus Biological), mouse anti human /mouse H2a-H2B Histone antibody 1µg/ml, (kind gift from Prof.Marc Monestier). Cells were washed in PBS, and further incubated with chicken anti rabbit IgG secondary antibody Alexa Fluor 594 (Life Technologies),and chicken anti mouse IgG secondary antibody Alexa Fluor 647 for 40 minutes, washed in PBS and mounted in Prolong Gold DAPI for identification of nuclear material (Life Technologies). Images were captured using an upright inverted Nikon C1 laser scanning microscope (Nikon, Tokyo Japan). Analysis was performed using IMARIS imaging analysis software (Bitplane AG, Zurich,Switzeralnd). The SURPASS select tool was used to select cells extruding extracellular traps (ETs determined as extracellular chromatin detected by DAPI or histones with co-expression of proteases) and to measure the individual cell ROS/MMP12 for macrophages and ROS/NE for neutrophils, expressed as arbitrary units (a.u). For comparison of MMP12, NE and ROS values between infected, non-infected and DNase treated groups, analysis was carried out on a minimum of 100 cells per sample, averaged and expressed as arbitrary units.

To define the correlation between ROS production and METs we used the ROS inhibitor apocynin, to inhibit the production of ROS (15)^.^ Macrophages were incubated with apocynin at a concentration of 100 µmol/litre for 20 minutes then NTHi added for 3 hours. As described above cells where then fixed and analysed using confocal microscopy and IMARIS for MET production by their extracellular production of chromatin using an established method [[12](#_ENREF_12)].

**1.9 Killing assay**

A primary function of macrophages is to kill phagocytosed bacteria. To test the ability of macrophages to kill bacteria we used a previously described method [[13](#_ENREF_13),[14](#_ENREF_14)]. Bacteria were incubated at a MOI of 100:1 to macrophages in 24-well plates for 2 hours (in antibiotic-free media). Wells were washed twice and Gentamicin (Sigma-Aldrich) added at a concentration of 100 μg per ml for one hour. Wells were then washed twice and sonicated for 20 seconds. Aliquots from duplicate wells were added to chocolate agar plates and cultured overnight. The next day the number of CFUs of NTHi was counted.

**1.10 Chemiluminescence**

The flow cytometry assay measures intracellular ROS. Most tissue damage arises from extracellular production of ROS. To measure extracellular production of ROS, L-O12-enhanced chemiluminescence was used as previously described [[15](#_ENREF_15)]. Briefly, BAL macrophages were seeded onto 96-well plates for 1-3 days. Cells were washed and then infected with strains of NTHi1-3 for one hour, after which time cells were exposed to L-O12 (100 mM). The production of ROS was then assessed by chemiluminescence with the Hidex Chameleon Microplate Reader at 37^0^C. Photon emission was recorded from each well every 2 min and averaged over 45 min. Individual data points for each group were derived from the average of 2 replicates. In some cases, cells were incubated with the cell-impermeable superoxide dismutase (SOD; 100 U/ml), which will inactivate superoxide and verify that the L-O12 chemiluminescence signal was due to extracellular superoxide [[16](#_ENREF_16)].

**1.11 In-vivo production of reactive oxygen species (ROS) in mouse lungs**

Using a well-established mouse model, BALB/c mice were infected intratracheally with 5x10^5^ colony forming units of NTHi-289 in 30 ml PBS or sham-infected with PBS vehicle as control as previously described [[17-19](#_ENREF_17)]. Five days after infection lungs were perfused, inflated, embedded in paraffin and sectioned (4-6 mm). This project was approved by the University of Newcastle animal ethics committee

A previously described method was used to measure 3-nitrotyrosine fluorescence.[[15](#_ENREF_15)] Lung sections were de-paraffinized with xylene, and hydrated in a series of graded ethanol solutions. Antigen retrieval was then performed in a boiling solution of 10 mM citrate buffer which was left to cool to room temperature. Tissue sections were washed in 0.01 M PBS ( pH 7.4; 3×10 min) before incubation in a mouse on mouse Ig blocking reagent (Vector Laboratories, Peterborough, United Kingdom) for 1 h to reduce non-specific binding. Sections were then incubated in mouse monoclonal anti-3-nitrotyrosine (1:50, AbCAM, Cambridge, MA, USA) overnight in a humid box. The following day, tissues were washed in 0.01 M PBS (3×10 min) to remove any excess antibody, and incubated in a biotinylated anti-mouse IgG reagent for 10 min for 3-nitrotyrosine studies. Lung sections were then washed in 0.01 M PBS (3×10 min) and Fluorescein Avidin DCS (Vector Laboratories) was applied for 5 min. Sections were washed in 0.01 M PBS (3×10 min) and cover slipped. Researchers were blinded throughout the experiment and all the appropriate primary and secondary controls were performed.

ROS production by 3-nitrotyrosine staining, was captured for each specimen in four 200x magnification images (area of 2 mm^2^), on a Nikon C1 confocal laser attached to a Nikon Ti-E inverted microscope using the 488nm laser. Each image was subsequently converted and analyzed using Image J (NIH, Bethseda, MD, USA). The intensity x density of immunofluorescence was measured and divided by the tissue area and reported as the average in arbitary units.

- 1. **Statistical analysis**

Statistical analysis was performed using Prism 6 software (GraphPad Software, San Diego, CA, USA). Comparison between control and stimulated groups was done using paired or unpaired-testing with parametric or non-parametric methods as appropriate. Between-group differences were analyzed by one-way analysis of the variance (ANOVA). A p value of less than 0·05 was considered to indicate statistical significance. Parametric results were represented graphically by mean and standard error of the mean, whilst non parametric reults were represented graphically by medians.

**SECTION 3: REFERENCES**

1. Rabe KF, Hurd S, Anzueto A, Barnes PJ, Buist SA, et al. (2007) Global strategy for the diagnosis, management, and prevention of chronic obstructive pulmonary disease: GOLD executive summary. Am J Respir Crit Care Med 176: 532-555.

2. Murphy TF (2005) Haemophilus infections. In: Braunwald F, Kaspar, Hauser, Longo, Jameson, editor. Harrisons Principles of Internal Medicine. 16 th ed. New York: McGraw Hill. pp. 864-866.

3. Moller LV, Timens W, van der Bij W, Kooi K, de Wever B, et al. (1998) Haemophilus influenzae in lung explants of patients with end-stage pulmonary disease. Am J Respir Crit Care Med 157: 950-956.

4. Dromann D, Rupp J, Rohmann K, Osbahr S, Ulmer AJ, et al. (2010) The TGF-beta-pseudoreceptor BAMBI is strongly expressed in COPD lungs and regulated by nontypeable Haemophilus influenzae. Respiratory research 11: 67.

5. Hodge S, Hodge G, Jersmann H, Matthews G, Ahern J, et al. (2008) Azithromycin improves macrophage phagocytic function and expression of mannose receptor in chronic obstructive pulmonary disease. Am J Respir Crit Care Med 178: 139-148.

6. Marti-Lliteras P, Regueiro V, Morey P, Hood DW, Saus C, et al. (2009) Nontypeable Haemophilus influenzae clearance by alveolar macrophages is impaired by exposure to cigarette smoke. Infect Immun 77: 4232-4242.

7. Thomas BJ, Lindsay M, Dagher H, Freezer NJ, Li D, et al. (2009) Transforming growth factor-beta enhances rhinovirus infection by diminishing early innate responses. Am J Respir Cell Mol Biol 41: 339-347.

8. King PT, Lim S, Pick A, Ngui J, Prodanovic Z, et al. (2013) Lung T-cell responses to nontypeable Haemophilus influenzae in patients with chronic obstructive pulmonary disease. The Journal of allergy and clinical immunology 131: 1314-1321 e1314.

9. King PT, Hutchinson PE, Johnson PD, Holmes PW, Freezer NJ, et al. (2003) Adaptive immunity to nontypeable Haemophilus influenzae. Am J Respir Crit Care Med 167: 587-592.

10. King PT, Hutchinson P, Holmes PW, Freezer NJ, Bennett-Wood V, et al. (2006) Assessing immune function in adult bronchiectasis. Clin Exp Immunol 144: 440-446.

11. Kuhns DB, Alvord WG, Heller T, Feld JJ, Pike KM, et al. (2010) Residual NADPH oxidase and survival in chronic granulomatous disease. The New England journal of medicine 363: 2600-2610.

12. Brinkmann V, Reichard U, Goosmann C, Fauler B, Uhlemann Y, et al. (2004) Neutrophil extracellular traps kill bacteria. Science 303: 1532-1535.

13. Ahren IL, Karlsson E, Forsgren A, Riesbeck K (2002) Comparison of the antibacterial activities of ampicillin, ciprofloxacin, clarithromycin, telithromycin and quinupristin/dalfopristin against intracellular non-typeable Haemophilus influenzae. J Antimicrob Chemother 50: 903-906.

14. King P, Ngui J, Oppedisano F, Robins-Browne R, Holmes P, et al. (2008) Effect of interferon gamma and CD40 ligation on intracellular monocyte survival of nontypeable Haemophilus influenzae. APMIS 116: 1043-1049.

15. Vlahos R, Stambas J, Bozinovski S, Broughton BR, Drummond GR, et al. (2011) Inhibition of Nox2 oxidase activity ameliorates influenza A virus-induced lung inflammation. PLoS pathogens 7: e1001271.

16. Dikalov S, Griendling KK, Harrison DG (2007) Measurement of reactive oxygen species in cardiovascular studies. Hypertension 49: 717-727.

17. Essilfie AT, Simpson JL, Dunkley ML, Morgan LC, Oliver BG, et al. (2012) Combined Haemophilus influenzae respiratory infection and allergic airways disease drives chronic infection and features of neutrophilic asthma. Thorax 67: 588-599.

18. Essilfie AT, Simpson JL, Horvat JC, Preston JA, Dunkley ML, et al. (2011) Haemophilus influenzae infection drives IL-17-mediated neutrophilic allergic airways disease. PLoS pathogens 7: e1002244.

19. Horvat JC, Starkey MR, Kim RY, Phipps S, Gibson PG, et al. (2010) Early-life chlamydial lung infection enhances allergic airways disease through age-dependent differences in immunopathology. The Journal of allergy and clinical immunology 125: 617-625, 625 e611-625 e616.
